# MitoNeoD: A Mitochondria-Targeted Superoxide Probe

**DOI:** 10.1016/j.chembiol.2017.08.003

**Published:** 2017-10-19

**Authors:** Maria M. Shchepinova, Andrew G. Cairns, Tracy A. Prime, Angela Logan, Andrew M. James, Andrew R. Hall, Sara Vidoni, Sabine Arndt, Stuart T. Caldwell, Hiran A. Prag, Victoria R. Pell, Thomas Krieg, John F. Mulvey, Pooja Yadav, James N. Cobley, Thomas P. Bright, Hans M. Senn, Robert F. Anderson, Michael P. Murphy, Richard C. Hartley

**Affiliations:** 1 WestCHEM School of Chemistry, University of Glasgow, Glasgow G12 8QQ, UK; 2 MRC Mitochondrial Biology Unit, University of Cambridge, Hills Road, Cambridge CB2 0XY, UK; 3 Department of Medicine, University of Cambridge, Addenbrooke's Hospital, Hills Road, Cambridge CB2 0QQ, UK; 4 School of Chemical Sciences, The University of Auckland, Private Bag 92019, Auckland 1142, New Zealand; 5 Division of Sport and Exercise Sciences, Abertay University, Dundee DD1 1HG, UK

**Keywords:** mitochondria, superoxide, ROS measurement, mitochondria-targeting, triphenylphosphonium, MitoSOX, hydroethidine, exomarker

## Abstract

Mitochondrial superoxide (O_2_^⋅−^) underlies much oxidative damage and redox signaling. Fluorescent probes can detect O_2_^⋅−^, but are of limited applicability *in vivo*, while in cells their usefulness is constrained by side reactions and DNA intercalation. To overcome these limitations, we developed a dual-purpose mitochondrial O_2_^⋅−^ probe, MitoNeoD, which can assess O_2_^⋅−^ changes *in vivo* by mass spectrometry and *in vitro* by fluorescence. MitoNeoD comprises a O_2_^⋅−^-sensitive reduced phenanthridinium moiety modified to prevent DNA intercalation, as well as a carbon-deuterium bond to enhance its selectivity for O_2_^⋅−^ over non-specific oxidation, and a triphenylphosphonium lipophilic cation moiety leading to the rapid accumulation within mitochondria. We demonstrated that MitoNeoD was a versatile and robust probe to assess changes in mitochondrial O_2_^⋅−^ from isolated mitochondria to animal models, thus offering a way to examine the many roles of mitochondrial O_2_^⋅−^ production in health and disease.

## Highlights

• MitoNeoD is a mitochondria-targeted O_2_^⋅−^ probe that can be used *in vivo*
• Neopentyl groups prevent DNA intercalation by MitoNeoD and its derivatives
• Incorporation of a carbon-deuterium bond enhances O_2_^⋅−^ selectivity by MitoNeoD
• MitoNeoD extends methods available to assess mitochondrial O_2_^⋅−^

## Graphical Abstract

**Figure:**
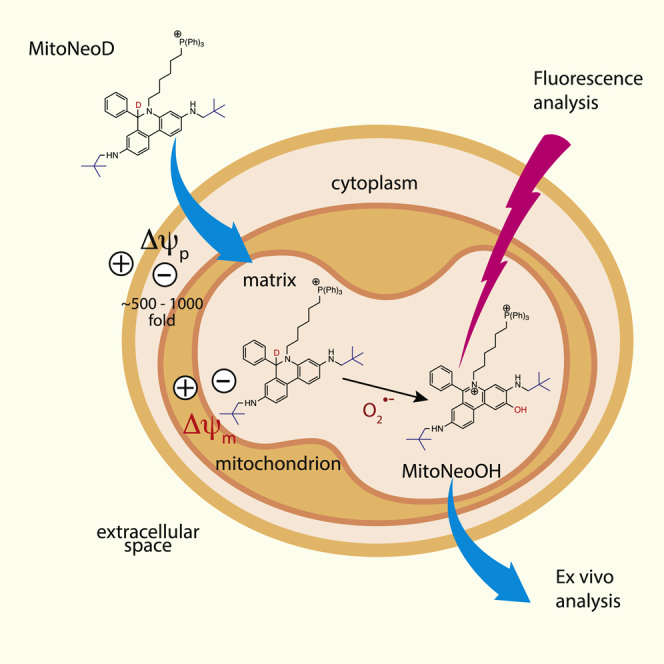

## Teaser

Current methods to assess mitochondrial O_2_^⋅−^ cannot be applied *in vivo* and are artifact prone. Here Shchepinova et al. introduce MitoNeoD, which can be used to assess changes in mitochondrial O_2_^⋅−^ by fluorescence and by mass spectrometry.

## Introduction

The production of O_2_^⋅−^ within the mitochondrial matrix varies under different conditions (Murphy, 2009, Winterbourn, 2008). Matrix O_2_^⋅−^ can initiate oxidative damage and can also dismutate to hydrogen peroxide (H_2_O_2_), which initiates redox signaling (Fourquet et al., 2008, Holmstrom and Finkel, 2014). Consequently, there is considerable interest in assessing the causes and effects of mitochondrial O_2_^⋅−^ production *in vitro* and *in vivo*; however, progress is hampered by the technical difficulties of assessing O_2_^⋅−^ (Halliwell and Whiteman, 2004, Kalyanaraman et al., 2014, Murphy et al., 2011).

Fluorescent probes based on hydroethidine (HE, dihydroethidium) such as HE, the mitochondria-targeted derivative MitoSOX Red (Robinson et al., 2006, Zielonka and Kalyanaraman, 2010), and the membrane-impermeant analogue hydropropidine (Michalski et al., 2013) are used to assess O_2_^⋅−^. Initially it was thought that HE was oxidized by O_2_^⋅−^ to the fluorescent product ethidium (E^+^), but Kalyanaraman and colleagues then showed that HE reacts with O_2_^⋅−^ to form 2-hydroxyethidium (2-OH-E^+^) (Zhao et al., 2003), while the production of E^+^ from HE can arise from many oxidants (Zielonka and Kalyanaraman, 2010). HE is first oxidized by O_2_^⋅−^ to a radical cation, which then reacts with another O_2_^⋅−^ to form a hydroperoxide adduct that rearranges to 2-OH-E^+^ (Figure S1) (Michalski et al., 2013, Michalski et al., 2014). However, the radical cation can also be formed by reaction with other oxidants, followed by further oxidation (or disproportionation) to E^+^ (Figure S1). Therefore, while the formation of 2-OH-E^+^ from HE is a robust indication of O_2_^⋅−^ generation, production of E^+^ from HE is not. Unfortunately, as the fluorescence of E^+^ and 2-OH-E^+^ overlap, and as E^+^ is often formed to a greater extent than 2-OH-E^+^, the assessment of O_2_^⋅−^ in cells through fluorescence by microscopy or flow cytometry is susceptible to artifact (Zielonka and Kalyanaraman, 2010). Therefore, to assess O_2_^⋅−^ production reliably the E^+^ and 2-OH-E^+^ products of HE and its derivatives have to be separated by high-pressure liquid chromatography (HPLC) followed by detection by fluorescence or mass spectrometry (Kalyanaraman et al., 2014, Maghzal and Stocker, 2007, Michalski et al., 2014, Zielonka and Kalyanaraman, 2010).

Another factor affecting the fluorescence of E^+^ and 2-OH-E^+^ is that both intercalate into DNA and double-stranded RNA (Horobin et al., 2013), thereby increasing the fluorescence quantum yield by 10- to 40-fold (Zhao et al., 2005, Zielonka et al., 2008) (Michalski et al., 2013) (Figure S1). The intercalation of the phenanthridinium oxidation products, E^+^ and 2-OH-E^+^, into DNA increases fluorescence in regions of the cell where DNA is abundant not just where O_2_^⋅−^ generation is elevated. This relocation from the cytosol to the nucleus (Horobin et al., 2013, Meany et al., 2007) and intercalation also makes such phenanthridinium salts toxic to DNA (Hashiguchi and Zhang-Akiyama, 2009). For these reasons, it would be good to separate the O_2_^⋅−^-sensing capabilities of HE analogues from their problematic interaction with DNA (Cairns et al., 2014).

**Figure 1:**
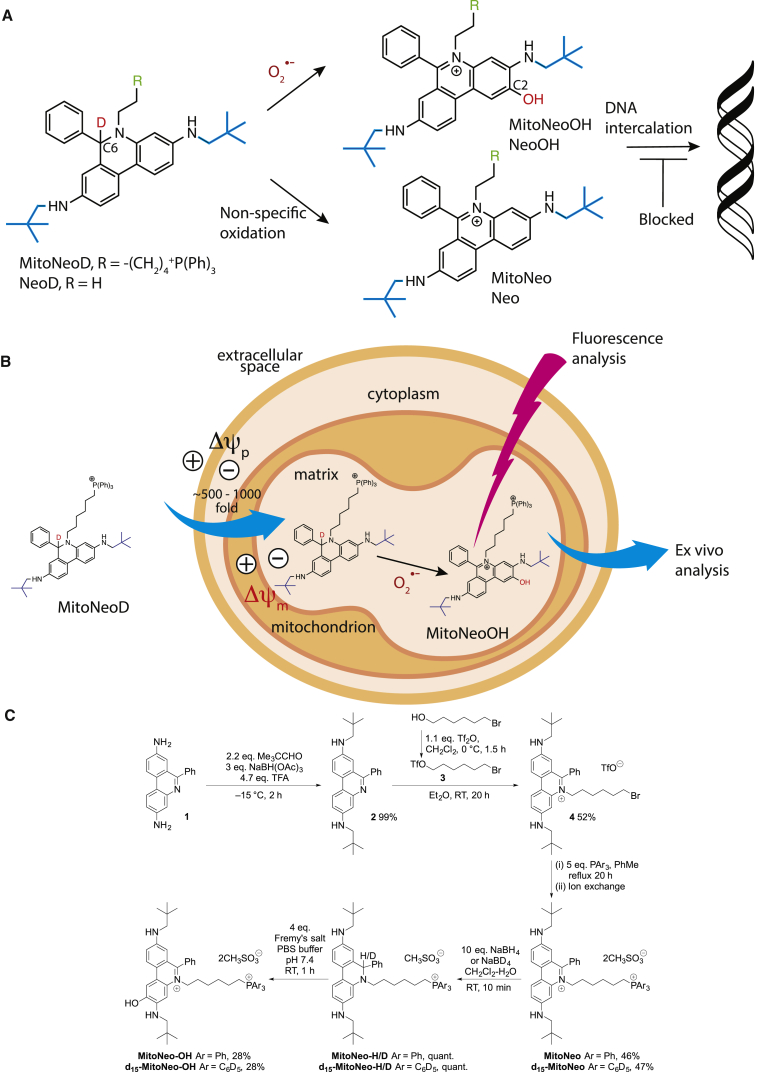
Selective Detection of O_2_^⋅−^ Using MitoNeoD (A) Reaction of MitoNeoD/H or NeoD/H with O_2_^⋅−^ generates MitoNeoOH or NeoOH, while non-specific oxidation forms MitoNeo or Neo. The bulky neopentyl groups (blue) prevent intercalation into DNA. (B) MitoNeoD uptake by mitochondria and reaction with O_2_^⋅−^. The membrane potential-dependent uptake of MitoNeoD by mitochondria in cells is shown, followed by its O_2_^⋅−^-dependent reaction to form MitoNeoOH, while the non-specific oxidation product MitoNeo is also formed. MitoNeoOH formation can be detected by confocal microscopy, or by extraction followed by LC-MS/MS to allow detection of mitochondrial O_2_^⋅−^*in vivo*. (C) Synthesis of MitoNeo, MitoNeoH/D, and MitoNeoOH. 3,8-Diamino-6-phenylphenanthridine **1** underwent double reductive amination with pivalaldehyde in the presence of sodium triacetoxyborohydride and trifluoroacetic acid (TFA) to give the bis(neopentyl) derivative **2**. Selective N*-*alkylation of the phenanthridine nitrogen atom was achieved using 6-bromohexyl triflate **3** to afford N*-*(bromohexyl)phenanthridinium salt **4**, which was then reacted with triphenylphosphine in toluene under reflux to furnish MitoNeo. d_15_-MitoNeo was prepared from N*-*(bromohexyl)phenanthridinium salt **4** in the same way using d_15_-triphenylphosphine. Reduction of MitoNeo in a two-phase water-dichloromethane mixture under argon by NaBH_4_/NaBD_4_ gave MitoNeoH/D. MitoNeoOH was prepared by reaction of MitoNeoH/D with Fremy's salt (potassium nitrosodisulfonate) in acetonitrile (ACN) and pH 7.4 phosphate buffer. See also Figures S1 and S2.

To overcome these limitations, we modified HE to develop a mitochondria-targeted O_2_^⋅−^ probe designed to produce phenanthridinum oxidation products that would not intercalate into DNA, and which could be used *in vivo*. Prevention of DNA intercalation was achieved by attaching bulky neopentyl groups to the 3- and 8-amino substituents on the phenanthridinium core (Figure 1A). Targeting to mitochondria was achieved by conjugation to the lipophilic triphenylphosphonium (TPP) cation, which drives the accumulation of drugs and probes into mitochondria in response to the membrane potential in cells and *in vivo* (Smith et al., 2011, Smith et al., 2012) (Figure 1B). To enhance probe stability in the presence of air and light, and to increase O_2_^⋅−^ selectivity, we also incorporated a deuterium at C-6 (Kundu et al., 2010). The rationale is that the oxidation of HE to either 2-OH-E^+^ or E^+^ requires cleavage of the C-H bond at C-6 on the hydrophenanthridine core of HE (Figure S1). Breaking this bond is the rate-limiting step of the spontaneous oxidation of HE to E^+^, probably occurring by hydrogen atom transfer, leading to a large kinetic isotope effect (KIE) of ∼4.7 (Kundu et al., 2010). In contrast, this C-H/D bond contributes less to the rate-limiting step for the reaction of HE with O_2_^⋅−^, giving a KIE of 2.5 (Kundu et al., 2010). Therefore deuterium incorporation should decrease background oxidation more than the O_2_^⋅−^-specific reaction, thereby increasing selectivity for O_2_^⋅−^ (Kundu et al., 2010). The precursor to our O_2_^⋅−^ probe, MitoNeo (Figure 1A), can be chemically reduced to the O_2_^⋅−^-sensitive probe MitoNeoD prior to use. While most O_2_^⋅−^ generated within mitochondria will most likely be converted to H_2_O_2_ by the action of Mn superoxide dismutase (SOD), the remaining small pool of O_2_^⋅−^ should react selectively with MitoNeoD to generate MitoNeoOH (Figure 1A), with non-specific oxidation generating MitoNeo (Figure 1A). The untargeted analogue, Neo, was also made and can be similarly reduced to NeoD (Figure S2A), which should react with O_2_^⋅−^ to generate NeoOH, reporting on O_2_^⋅−^-production in the cytosol (Figure S2B).

To extend MitoNeoD to assess the production of O_2_^⋅−^ by mitochondria *in vivo* we used the exomarker approach (Logan et al., 2014). For this, the probe is administered *in vivo* and there reacts with the species of interest to form an exomarker that is extracted and assayed *ex vivo* by mass spectrometry (Cochemé et al., 2011, Cochemé et al., 2012, Logan et al., 2014, Pun et al., 2014) (Figure 1B). This approach enables changes in the levels of reactive species within the mitochondria *in vivo* to be determined. We have done this previously for MitoB, in which a TPP moiety is used to target a H_2_O_2_- and peroxynitrite-reactive boronic acid moiety to mitochondria, which there reacts to form a diagnostic exomarker MitoP (Cochemé et al., 2011, Cochemé et al., 2012). The MitoP/MitoB ratio can then be assessed *ex vivo* to infer changes in the concentration of these reactive species within the mitochondria of a living organism. To facilitate quantification, these species were measured relative to deuterated internal standards by liquid chromatography-tandem mass spectrometry (LC-MS/MS) after extraction from the tissue. A further advantage of the TPP cation is that its fixed positive charge greatly enhances the sensitivity for compound detection by mass spectrometry, enabling the measurement of pmol/g levels in tissue, while the ratiometric measurement of MitoP and MitoB corrects for changes in uptake *in vivo* (Cochemé et al., 2011, Cochemé et al., 2012). By extension, we can inject MitoNeoD into a living organism, where it should be taken up into mitochondria, and converted by O_2_^⋅−^ to MitoNeoOH. Subsequent extraction of the tissue *ex vivo* and quantification of the amounts of MitoNeo and MitoNeoOH by LC-MS/MS relative to deuterated internal standards should enable changes in formation of mitochondrial O_2_^⋅−^ *in vivo* to be assessed. Here we report on the development of MitoNeoD and show that it enables the detection of mitochondrial O_2_^⋅−^ by fluorescence and by mass spectrometry in cells and *in vivo* (Figure 1B).

## Results

### Synthesis and Properties of MitoNeoD

MitoNeoD (Figure 1A) incorporates bulky neopentyl groups to prevent DNA intercalation, while having similar electron-donating properties to the 3- and 8-amino substituents of HE, which ensures high reactivity with O_2_^⋅−^ and prevents ring opening of the oxidation products to form pseudobases (Bunting and Meathrel, 1974). The neopentylamino groups are also more acid-stable than tertiary butyl derivatives and do not sterically impede the reaction with O_2_^⋅−^. These electronic and steric properties played a significant role in the chemical synthesis of MitoNeoD (Figure 1C). The neopentyl groups were introduced by reductive amination of 3,8-diamino-6-phenylphenanthridine **1** with pivalaldehyde giving a relatively electron-rich phenanthridine **2**. This allowed selective N*-*alkylation of the phenanthridine in the presence of the arylamino groups to furnish the bromo-derivative **4**. Displacement of the bromide by triphenylphosphine or d_15_-triphenylphosphine afforded MitoNeo and d_15_-MitoNeo, respectively, the latter of which is required for LC-MS/MS quantification (Cochemé et al., 2011).

MitoNeo is reduced to MitoNeoH/D by sodium borohydride/borodeuteride (Figure 1C), and it is this hydrophenanthridine/deuterophenanthridine form that will be used to assess O_2_^⋅−^ formation. MitoNeoH/D should react selectively with O_2_^⋅−^ to form MitoNeoOH, and non-specifically with other oxidants to form MitoNeo (Figure 1A). MitoNeoOH is produced selectively because the transformation requires two distinct features of O_2_^⋅−^: its reactivity as a nucleophilic radical and the presence of a hydroxide leaving group in the resulting adduct. The synthetic compound, Fremy's salt (potassium nitrosodisulfonate), which shares these two characteristics and mimics the chemistry of the O_2_^⋅−^ reaction (Zielonka et al., 2008), was used to prepare a definitive sample of MitoNeoOH from MitoNeoH (Figure 1C). We also report the synthesis of NeoH/D and the corresponding oxidation products NeoOH and Neo (Figure S2A), which will not be targeted to mitochondria and should instead report on O_2_^⋅−^ production in the cytosol (Figure S2B).

**Figure 2:**
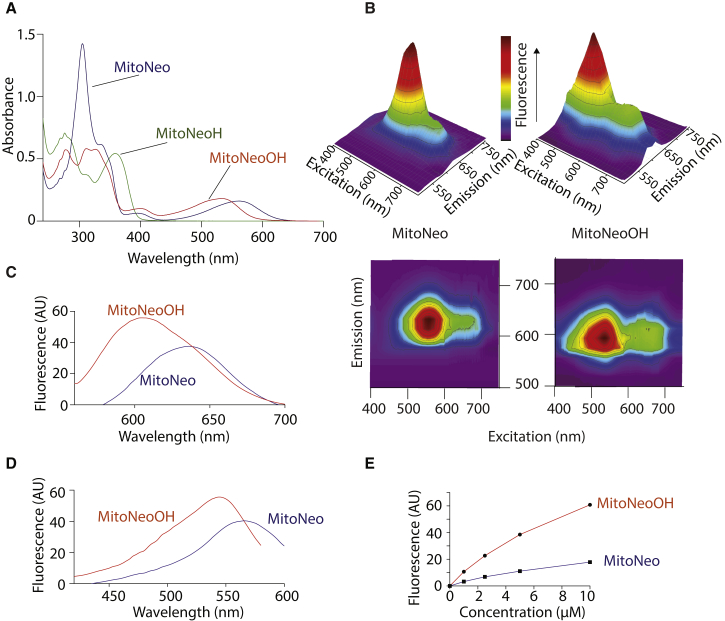
Optical Properties of MitoNeo and Its Derivatives (A) UV-visible spectra of MitoNeo, MitoNeoOH, and MitoNeoH (25 μM of each) in ethanol. (B–D) Fluorescence spectra of MitoNeo and/or MitoNeoOH (25 μM of each in KCl buffer). (B) Fluorescence spectra of MitoNeo and/or MitoNeoOH as a function of excitation and emission wavelength, shown in 3D (upper) and 2D (lower) views. (C) Emission fluorescence spectra of MitoNeo and MitoNeoOH. Excitation wavelengths: 566 nm (MitoNeo), 544 nm (MitoNeoOH). (D) Excitation fluorescence spectra of MitoNeo and MitoNeoOH. Emission wavelengths: 636 nm (MitoNeo), 605 nm (MitoNeoOH). (E) Concentration dependence of fluorescence of MitoNeoOH and MitoNeo at the excitation (544 nm) and emission (605 nm) maxima of MitoNeoOH. See also Figure S3 and Table S1.

The UV-visible absorption spectra of the MitoNeo and Neo compounds are shown in Figures 2A, S3A, and S3B. There is minimal interference of MitoNeoH absorption with MitoNeo/MitoNeoOH above ∼400 nm, and significant MitoNeoH absorption at ∼385 nm, where there is minimal absorption by the oxidized forms. The local absorption maxima and extinction coefficients are given in Table S1.

The fluorescence of MitoNeo and MitoNeoOH is shown in Figure 2B. MitoNeoH/D are not fluorescent at these wavelengths. The excitation and emission maxima for MitoNeo are in the range of 540–580 and 600–650 nm, respectively, and for MitoNeoOH are in the range of 520–560 and 580–620 nm, respectively. To refine these assignments, we assessed the emission (Figure 2C) and excitation (Figure 2D) spectra for MitoNeo and MitoNeoOH, from which we could infer excitation/emission maxima of 566/636 nm for MitoNeo and 544/605 nm for MitoNeoOH (Table S1). The emission (Figures S3C and S3D; Table S1) and excitation (Figure S3E; Table S1) spectra for Neo and NeoOH are similar. The negligible MitoNeoD fluorescence may facilitate monitoring of the oxidation of MitoNeoD to MitoNeoOH, and excitation at shorter wavelength should minimize interference from the non-specific oxidation product, MitoNeo. To determine if this is the case we assessed the relative fluorescence of MitoNeo and MitoNeoOH at the excitation/emission maxima for MitoNeoOH (544/605 nm) (Figure 2E), which showed that the fluorescence increase with concentration is four times greater for MitoNeoOH than for MitoNeo, with similar selectivity for NeoOH over Neo (Figure S3F). Therefore fluorescence changes are partially selective for the O_2_^⋅−^-sensitive reaction of MitoNeoD.

### Analysis of MitoNeoH/D Reactivity with O_2_^⋅−^

**Figure 3:**
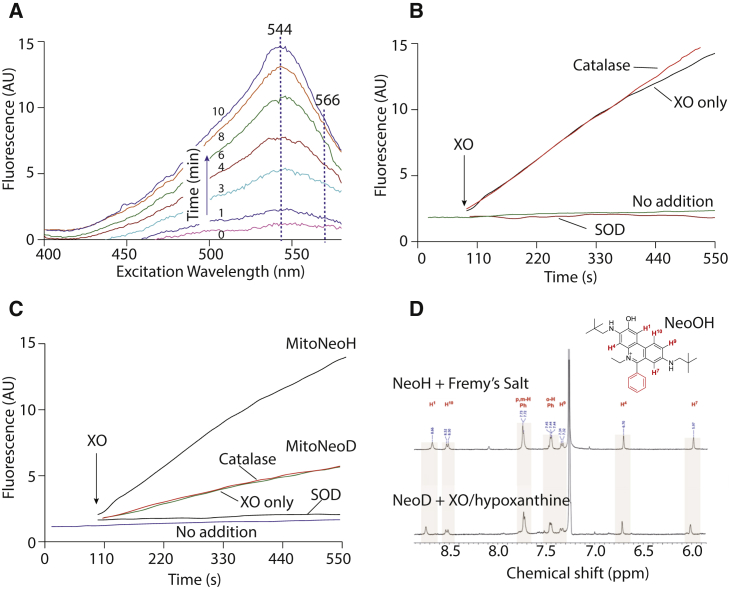
Fluorescence and NMR Analysis of Reaction of MitoNeoH/D with O_2_^⋅−^ (A) Excitation fluorescence spectra over time. MitoNeoH (10 μM) was incubated at 37°C in KCl buffer with 1 mM hypoxanthine (HX) and 5 mU/mL xanthine oxidase (XO) and the excitation spectrum was assessed at various times using an emission wavelength of 605 nm. (B and C). Time courses of reaction of MitoNeoH (B) or MitoNeoD (C) with O_2_^⋅−^. MitoNeoH or MitoNeoD (10 μM) was incubated with 1 mM HX and 5 mU/mL XO, in the presence of 10 μg/mL SOD or 50 U/mL catalase in KCl buffer at 37°C. Excitation and emission wavelengths were 544 and 605 nm, respectively. (D) ^1^H NMR analysis of reaction product of NeoD with O_2_^⋅−^. The upper ^1^H NMR spectrum is of NeoOH in CDCl_3_, synthesized from NeoH using Fremy's salt. For the lower spectrum, NeoD (100 μM) was exposed to O_2_^⋅−^ by incubation with XO (0.5 U/mL) and HX (1 mM) for 3 hr in a 1:0.5:3.5 mixture of EtOH:PBS:H_2_O and then extracted into CHCl_3_, purified by HPLC and the ^1^H NMR spectrum obtained. The expansion is of the aromatic region of the spectrum where only the numbered protons of the phenanthridinium moiety and those on the 6-phenyl group appear (red on the MitoNeoOH structure). See also Figure S4.

We next assessed the reaction of MitoNeoH with O_2_^⋅−^, generated by hypoxanthine/xanthine oxidase. The fluorescence excitation spectrum of MitoNeoOH increased over time upon exposure to O_2_^⋅−^ (Figure 3A). The excitation maximum was similar to that for MitoNeoOH (544 nm) rather than that for MitoNeo (566 nm), consistent with the reaction primarily generating MitoNeoOH. We then assessed the changes in fluorescence over time at the optimal wavelengths for MitoNeoOH (544/605 nm; Figure 3B). This showed little spontaneous oxidation of MitoNeoH, while there was a dramatic increase on exposure to O_2_^⋅−^. This increase was blocked by degrading O_2_^⋅−^ with SOD, but not by intercepting H_2_O_2_ with catalase (Figure 3B). To see how incorporation of a deuterium atom at C-6 affected oxidation, we exposed MitoNeoD to O_2_^⋅−^ (Figure 3C). MitoNeoH and MitoNeoD were qualitatively the same; however, the fluorescence of the oxidation products of MitoNeoD increased ∼2.9 times more slowly than MitoNeoH (Figure 3C). NeoH exposed to O_2_^⋅−^ also showed oxidation consistent with the formation of NeoOH (Figures S4A–S4C), with NeoD being oxidized approximately half as fast as NeoH (Figure S4D). These data are consistent with the O_2_^⋅−^-specific oxidation of MitoNeoD to MitoNeoOH.

To determine whether the NeoOH/MitoNeoOH synthesized using Fremy's salt are the same as the products of the reaction of MitoNeoD or NeoD with O_2_^⋅−^ and to confirm the hydroxyl location in MitoNeoOH/NeoOH, we analyzed the product of the reaction of NeoD with O_2_^⋅−^ by nuclear magnetic resonance (NMR) (Figures 3D and S4E–S4I). The upper ^1^H NMR spectrum in Figure 3D is of authentic NeoOH, synthesized from NeoH using Fremy's salt (Figure S2A). When NeoD was exposed to O_2_^⋅−^ the ^1^H NMR spectrum (Figure 3D, lower spectrum) was essentially identical to that of authentic NeoOH. Together, these data are consistent with the O_2_^⋅−^-dependent selective oxidation of MitoNeoD to MitoNeoOH.

### Reverse-Phase HPLC Analysis of Reactions of MitoNeoD with O_2_^⋅−^ and Other Reactive Oxygen Species

**Figure 4:**
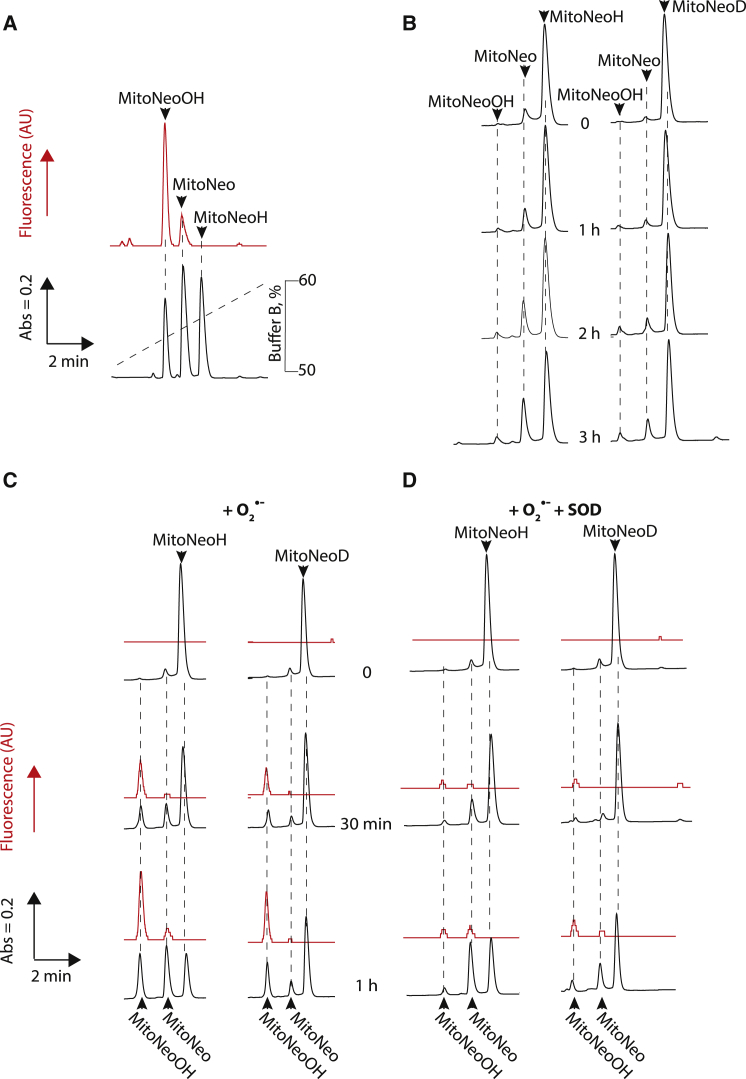
RP-HPLC Analysis of MitoNeoD Reaction with O_2_^⋅−^ (A) RP-HPLC chromatogram of a mixture of MitoNeoH, MitoNeo, and MitoNeoOH (10 nmol of each), assessed by absorbance at 220 nm and by fluorescence at the excitation (550 nm) and emission (590 nm) peaks for MitoNeoOH, under RP-HPLC conditions. (B) Stability of MitoNeoH and MitoNeoD. RP-HPLC of MitoNeoH or MitoNeoD (100 μM) incubated in KCl buffer at 37°C. At the indicated times, 10 nmol aliquots were removed and analyzed by RP-HPLC. (C) Reaction of MitoNeoH and MitoNeoD with O_2_^⋅−^. RP-HPLC of MitoNeoH or MitoNeoD (100 μM) incubated in KCl buffer at 37°C with 1 mM HX and 5 mU/mL XO. At the indicated times, 10 nmol aliquots were removed and analyzed by RP-HPLC. (D) Effect of SOD on the reaction of MitoNeoH and MitoNeoD with O_2_^⋅−^. RP-HPLC of MitoNeoH or MitoNeoD (100 μM) incubated in KCl buffer (pH 7.2) at 37°C with gentle shaking 1 mM HX and 5 mU/mL XO supplemented with 10 μg/mL SOD. At the indicated times, 10 nmol aliquots were removed and analyzed by RP-HPLC. See also Figure S5.

MitoNeo, MitoNeoH/D and MitoNeoOH can be separated by reverse-phase HPLC (RP-HPLC) (Figure 4A), so we used this to assess their relative stability and reactivity with O_2_^⋅−^. As anticipated, in the absence of O_2_^⋅−^ MitoNeoD was more resistant to spontaneous oxidation to MitoNeo than was MitoNeoH (Figure 4B), with ∼3-fold more oxidation of MitoNeoH compared with MitoNeoD, consistent with their relative KIEs. Incubation with O_2_^⋅−^ led to the formation of MitoNeoOH and MitoNeo over time (Figure 4C). Although MitoNeo was formed it contributed little to the fluorescence optimized for MitoNeoOH. Exposure of MitoNeoD to O_2_^⋅−^ led to more formation of MitoNeoOH relative to MitoNeo than for incubation with MitoNeoH (Figure 4C), with the ratio of the peak areas of MitoNeoOH to MitoNeo being ∼0.8 for MitoNeoH after an hour, while for MitoNeoD this was ∼2. In contrast, under background conditions (Figure 4B), the same ratios were ∼0.3 and 0.7, respectively. Degradation of O_2_^⋅−^ by SOD prevented the accumulation of MitoNeoOH, but there was still some MitoNeo formation (Figure 4D), and the MitoNeoOH/MitoNeo ratios were about the same as occurred during background oxidation. Exposure of MitoNeoD to other biologically relevant reactive oxygen species (ROS) showed negligible formation of MitoNeoOH (Figure S5A). Thus, MitoNeoD is more stable than MitoNeoH, the formation of MitoNeoOH only occurs in the presence of O_2_^⋅−^, and MitoNeoD is more selective for O_2_^⋅−^ than MitoNeoH.

### Reaction of MitoNeoH/D with O_2_^⋅−^ Measured by Pulse Radiolysis

To investigate the mechanism of the reaction of MitoNeoH/D with O_2_^⋅−^, the rates of the first one-electron oxidation step (Figure S1) were analyzed by pulse radiolysis. The spectrum of the radical cation formed by the one-electron oxidation of MitoNeoH in water-ethanol at pH 6 (Figure S5B), where the α-hydroxyethylperoxyl radical is the oxidizing species, is similar to the aniline radical cation, which absorbs in the 400–450 nm region (Qin et al., 1985); the shift to longer wavelengths for the radical cation of MitoNeoH is expected for N-substituted aniline radicals (Christensen, 1972). The one-electron oxidation of MitoNeoH in water-ethanol solution at pH 11, where O_2_^⋅−^ is the oxidizing species, generates the deprotonated anilino radical (pK_a_ 7.1), which absorbs in the same region as the aniline radical cation, but with much lower intensity (Figure S5B). The formation of these products at 475 nm was used to determine the rates of reaction with O_2_^⋅−^ (Figure S5B, inset): MitoNeoH = 1.25 ± 0.01 × 10^7^ M^−1^ s^−1^; MitoNeoD = 1.42 ± 0.04 × 10^7^ M^−1^ s^−1^ (Figure S5C). The reaction of NeoH and NeoD with O_2_^⋅−^ gave rate constants of: 1.08 ± 0.07 × 10^7^ and 1.09 ± 0.13 × 10^7^ M^−1^ s^−1^, respectively (Figure S5D). Hence, deuterium incorporation did not lead to a primary KIE. A previous assessment of the reaction of HE with O_2_^⋅−^ by pulse radiolysis in 1:1 water:ethanol at pH 8 gave *k* = 2 × 10^6^ M^−1^ s^−1^ (Zielonka et al., 2006), consistent with our results. However, these authors now favor far lower rates for this reaction (6 × 10^3^ M^−1^ s^−1^ for HE and 1.4 × 10^4^ M^−1^ s^−1^ for MitoSOX Red, based on a competition assay with SOD [Michalski et al., 2013]). These authors discounted the earlier pulse radiolysis measurements because the protonated form of O_2_^⋅−^, the perhydroxyl radical (HO_2_^⋅^), may have dominated the reaction. However, this is unlikely because the pK_a_ of HO_2_^⋅^ in water is 4.8 (Bielski et al., 1985). Our view is that both measurements are correct: the rate constants determined by the competition assay are for the overall reaction, while those determined by pulse radiolysis are for the first step only, which is not rate determining. This is consistent with our observations that there is no KIE for the first one-electron oxidation, but that the overall reaction with O_2_^⋅−^ is slower for MitoNeoD/NeoD than for MitoNeoH/NeoH (Figures 3B, 3C, S4C, and S4D). An overall rate of ∼10^4^ M^−1^ s^−1^ for the reaction of MitoNeoD with O_2_^⋅−^ is far less than the rate for the reaction of O_2_^⋅−^ with MnSOD (∼2 × 10^9^ M^−1^ s^−1^), hence MitoNeoD will report on O_2_^⋅−^ levels without distortion of its concentration.

The lack of a KIE by pulse radiolysis rules out hydrogen atom abstraction from the C-6 of MitoNeoH by O_2_^⋅−^ as the first step in the reaction. However, these data are consistent with one-electron oxidation by O_2_^⋅−^ forming a radical cation (Zielonka et al., 2006), as is shown in Figure S5E. There is then a larger KIE for the deuterium atom transfer from the radical intermediate to form MitoNeo, than for the alternative reaction sequence with O_2_^⋅−^ to form MitoNeoOH (Figure S5E), in which the C-6 deuterium atom is lost by deuteron transfer. Thus, MitoNeoD exhibits greater selectivity than MitoNeoH for the reaction with O_2_^⋅−^ to form MitoNeoOH over the competing oxidation to MitoNeo.

### MitoNeo and Neo Do Not Intercalate into DNA

**Figure 5:**
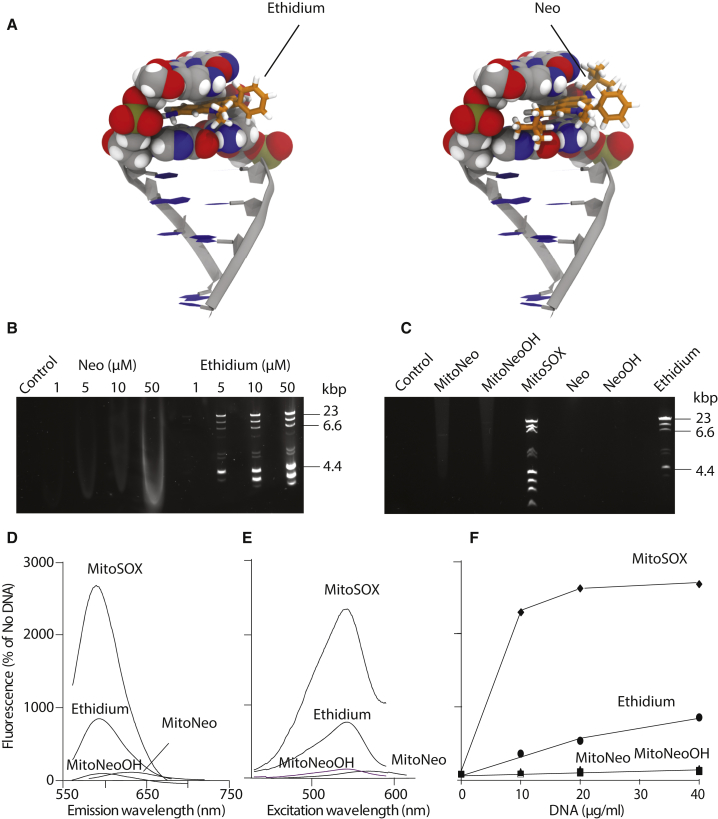
Lack of DNA Binding by MitoNeo and Its Derivatives (A) Models of the intercalation of ethidium (E^+^, left) and Neo (right) into DNA. The two molecules were docked into a rigid piece of DNA (d(CGTACG)) by manually replacing the intercalator present in the original X-ray structure and optimizing, starting with different orientations and rotamers for E^+^ and Neo. (B) A DNA ladder (10 μg) was mixed with the indicated concentrations of compounds, separated by electrophoresis on an agarose gel and visualized under UV transillumination. (C) A DNA ladder was incubated with 10 μM of the indicated compounds, separated and assessed as in (B). (D and E) Fluorescence emission (D) and excitation (E) spectra in the presence of DNA. MitoNeo, MitoNeoOH, MitoSOX or E^+^ (50 μM) were dissolved in KCl buffer. The spectra were measured and measured again after addition of 40 μg DNA, and are shown as percentage of the intensity in the absence of DNA. The excitation and emission wavelengths used were 566 and 636 nm (MitoNeo), 544 and 605 nm (MitoNeoOH), 526 and 605 nm (E^+^ and MitoSOX), respectively. (F) Dependence of fluorescence on DNA concentration. The fluorescence intensity of 50 μM of the indicated compounds was measured without DNA and again after sequential addition of the indicated amounts of DNA. Data are expressed as a percentage of the intensity in the absence of DNA and are a representative experiment of three replicates.

To assess whether the neopentyl groups on MitoNeo block intercalation into DNA, we first used a modeling approach that can be described as manual rigid docking. Starting from the X-ray structure of a 6-bp double-stranded DNA (d(CGTACG)) containing an acridine-based intercalator (Todd et al., 1999), we kept the nucleic acid scaffold fixed and manually replaced the intercalator with E^+^. Using energy minimization, we optimized the structure and position of the inserted E^+^. In doing this, two starting orientations of E^+^ were considered, obtained by flipping the molecule by 180°, with the substituents on the central ring sticking out into the major groove. Neo was docked in the same way, considering again two orientations. As shown in Figure 5A, E^+^ easily intercalates, whereas Neo cannot fit in-between the base pairs, due to the neopentyl groups. Therefore, Neo cannot insert deep enough between the DNA bases to allow for favorable π-π stacking interactions, consistent with the neopentyl groups decreasing the affinity of Neo for DNA compared with E^+^.

However, this simple model does not account for the flexibility of DNA, effects of solvent and salts, or for the influence of the TPP moiety of MitoNeo. Therefore we assessed the DNA binding of Neo and MitoNeo experimentally. To do this, we separated a DNA ladder by electrophoresis in the presence of Neo and E^+^ and then measured fluorescence (Figure 5B). This showed extensive E^+^ fluorescence associated with the DNA, but no localization of Neo fluorescence in DNA bands, consistent with negligible intercalation. A similar analysis showed that, while MitoSOX intercalated into DNA, MitoNeo, MitoNeoOH, and NeoOH did not (Figure 5C). The intercalation of E^+^ and of MitoSOX into DNA increases fluorescence by 20- to 25-fold for MitoSOX and 7- to 10-fold for E^+^ (Figures 5D, 5E, and 5F). In contrast, addition of DNA did not alter MitoNeo or MitoNeoOH fluorescence (Figures 5D, 5E, and 5F). In summary, the bulky neopentyl groups of MitoNeo and of its 2-hydroxy derivative MitoNeoOH prevents their intercalation into DNA.

### Uptake and Oxidation of MitoNeoD by Mitochondria and Cells

**Figure 6:**
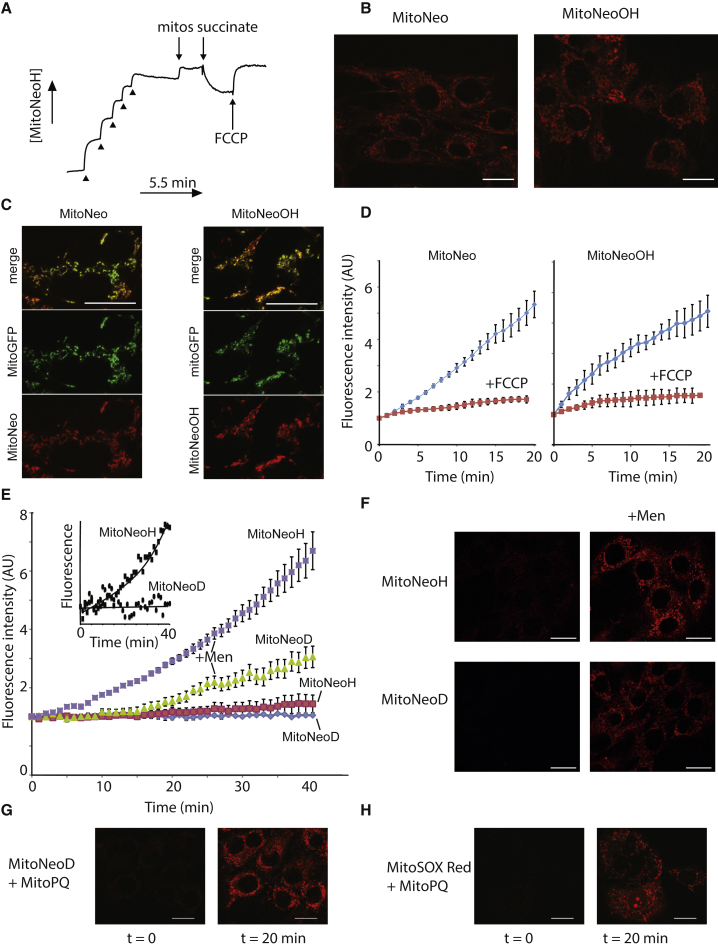
Uptake of MitoNeo Compounds by Mitochondria and Cells (A) Ion-selective electrode measurements of MitoNeoH uptake into isolated mitochondria. MitoNeoH (five consecutive 1 μM additions; arrowheads) was added to KCl buffer, followed by unenergized mitochondria (mitos), succinate, and FCCP. (B) Analysis of cell uptake of MitoNeo and MitoNeoOH by confocal microscopy. C2C12 cells were incubated with MitoNeo or MitoNeoOH (5 μM) as indicated and the fluorescence determined after 20 min. Scale bar, 20 μm. (C) Mitochondrial localization of MitoNeo by confocal microscopy. HEK-mitoGFP transgene cells were incubated with MitoNeo or MitoNeoOH (5 μM) for 10 min and then visualized by confocal microscopy. Scale bars, 10 μm. (D) Assessment of effect of FCCP on uptake of MitoNeo and MitoNeoOH into cells. Cells were incubated as in (B) in the presence and absence of FCCP, and the fluorescence in mitochondrial regions quantified four times and the means ± SD are plotted against time. (E) Oxidation of MitoNeoH and MitoNeoD in C2C12 cells. The oxidation of MitoNeoH and MitoNeoD in the absence of added oxidants, or in the presence of menadione (men, 500 nM). Data are the means ± SD of four replicates. The background levels of oxidation in the absence of oxidants is expanded in the inset. (F) Oxidation of MitoNeoD and MitoNeoH in C2C12 cells by menadione (men). Cells were imaged after 20 min incubation in the presence or absence of menadione (0.5 μM). Scale bars, 20 μm. (G) Oxidation of MitoNeoD in C2C12 cells by MitoPQ. Cells were incubated with MitoPQ (1 μM) for 20 min. Scale bars, 10 μm. (H) Oxidation of MitoSOX Red in C2C12 cells by MitoPQ. Cells were incubated with MitoPQ (1 μM) for 20 min. Scale bars, 10 μm. See also Figure S6.

To serve as a mitochondria-targeted probe, MitoNeoD must be accumulated by mitochondria in response to the membrane potential. MitoNeoD showed negligible impact on the function of isolated mitochondria at concentrations below 25 μM (Figure S6A). To assess uptake qualitatively in isolated mitochondria we first used an electrode responsive to the TPP cation (Kamo et al., 1979) (Figure 6A). This showed that energization of mitochondria with the respiratory substrate succinate led to uptake of MitoNeoH into mitochondria and that abolishing the membrane potential with the uncoupler carbonyl cyanide 4-(trifluoromethoxy)phenylhydrazone (FCCP) released MitoNeoH (Figure 6A).

We then assessed the uptake of MitoNeo by mitochondria within cells, first establishing non-toxic concentrations (Figures S6B and S6C) and determining how they affected respiration (Figure S6D). It was not possible to use fluorescence microscopy to measure the uptake of MitoNeoD within cells, because its fluorescence overlapped with endogenous autofluorescence. In contrast, the uptake of the highly fluorescent MitoNeo and MitoNeoOH by cells was readily observed and showed mitochondrial localization (Figure 6B). Similar experiments in cells expressing a mitochondria-targeted GFP (mitoGFP) confirmed that both MitoNeo and MitoNeoOH localized to mitochondria (Figure 6C). FCCP decreased the uptake of MitoNeo and MitoNeoOH into mitochondria (Figure 6D) and led to the slow release when added after MitoNeo or MitoNeoOH had accumulated for 10 min (Figure S6E).

To see if confocal fluorescence microscopy could differentiate between the formation of MitoNeoOH and MitoNeo, and thereby better assess mitochondrial O_2_^⋅−^ formation, we measured the change in fluorescence over time of MitoNeoOH or MitoNeo incubated with cells, using wavelengths optimized for the detection of MitoNeoOH (Figure S6F). This showed that there was enhanced sensitivity to MitoNeoOH over MitoNeo.

We next determined whether MitoNeoD could detect mitochondrial O_2_^⋅−^ production within cells by confocal fluorescence microscopy (Figures 6E and 6F). The background rates of oxidation of MitoNeoH or MitoNeoD by unstressed cells were low, with MitoNeoD being more stable (Figure 6E, inset). Increasing O_2_^⋅−^ production by the redox cycler menadione increased mitochondrial fluorescence for both MitoNeoH and MitoNeoD (Figures 6E and 6F). MitoNeoH was more sensitive to oxidation by O_2_^⋅−^ than MitoNeoD (Figure 6E). However, the oxidation of MitoNeoD is a more reliable readout of O_2_^⋅−^ levels than MitoNeoH, due to its lower sensitivity to non-specific oxidation to MitoNeo. MitoNeoD was oxidized by the mitochondria-targeted redox cycler MitoPQ, which generates O_2_^⋅−^ by redox cycling at complex I (Robb et al., 2015) (Figure 6G). MitoPQ also led to oxidation of MitoSOX Red generating fluorescence that was largely localized to the mitochondria, but with some labeling of nuclear DNA (Figure 6H). To assess this further we compared nuclear staining under more oxidizing conditions generated by 5 μM MitoPQ and found that MitoSOX Red stained most of the nuclei assessed (78% ± 20%, n = 4 independent cell fields ± SD) with no staining by MitoNeoD. This is expected as MitoNeo and its derivatives do not intercalate into DNA (Figures 5D–5F). We conclude that, while the increase in fluorescence upon oxidation of MitoNeoD is less than that for MitoSOX Red, it is a more selective indicator of mitochondrial O_2_^⋅−^ formation.

### Analysis of MitoNeoOH Formation by LC-MS/MS

**Figure 7:**
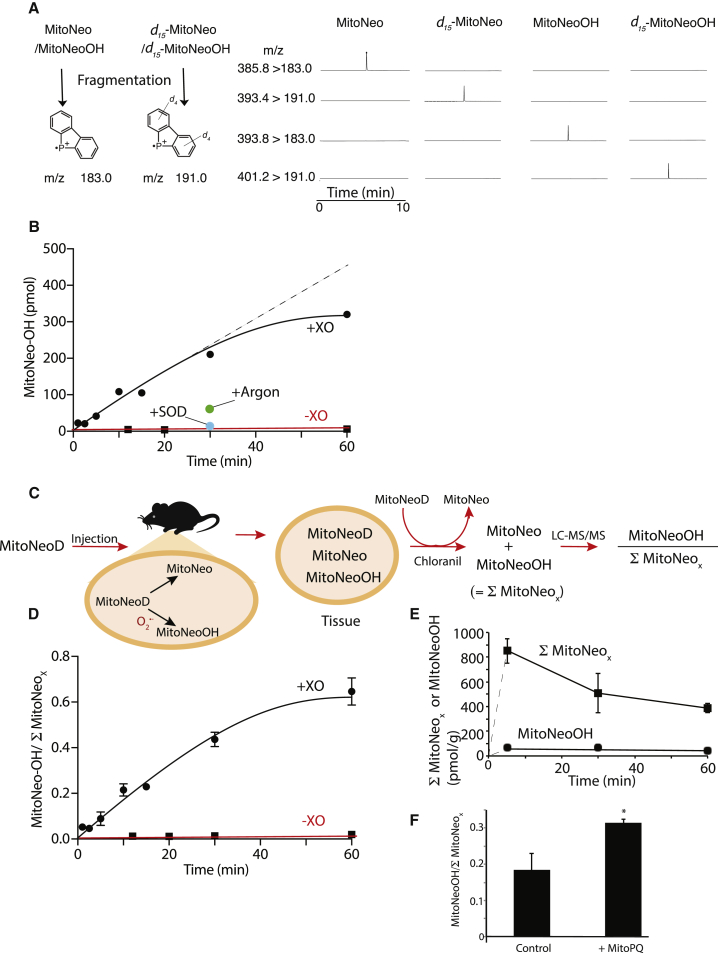
LC-MS/MS analysis of O_2_^⋅−^-Dependent MitoNeoOH Formation *In Vitro* and *In Vivo* (A) Typical LC-MS/MS chromatograms showing the m/z transitions measured simultaneously for 50 nM each of MitoNeo, *d*_*15*_-MitoNeo, MitoNeoOH, and *d*_*15*_-MitoNeoOH. Traces are normalized to the maximum total ion count for that experiment. (B) *In vitro* formation of MitoNeoOH over time. MitoNeoD (20 μM) was incubated with 1 mM HX and 5 mU/mL XO in 250 μL KCl buffer with shaking at 37°C for up to 60 min. The reaction was stopped by the addition of chloranil (10 μL of 10 mM) and incubated with shaking at 37°C for 30 min and extracted and analyzed by LC-MS/MS. Where indicated, the incubations were bubbled with argon, or SOD (20 μg/mL) was added. Data are n = 3 ± SEM for three incubations. (C) Schematic of oxidation of MitoNeoD by chloranil, followed by extraction. (D) Change of MitoNeoOH//Σ MitoNeo_×_ over time. Samples from the incubation described in (B) were examined further by measuring the content of MitoNeo by LC-MS/MS, enabling the MitoNeoOH//Σ MitoNeo ratio to be calculated. (E) Uptake of MitoNeo compounds into the heart *in vivo.* MitoNeoD (25 nmol) was given via tail vein injection. After 5, 30, or 60 min, hearts were removed and immediately snap frozen. Tissue (50 mg) was then extracted in the presence of chloranil, enabling MitoNeo and MitoNeoOH levels to be assessed by LC-MS/MS. The combined levels of MitoNeoD, MitoNeo, and MitoNeoOH are shown as Σ MitoNeo_X_. The contribution of MitoNeoOH alone to Σ MitoNeo_X_ is also shown. Dashed lines are interpolations from t = 0 to the first data point. Data are n = 3 ± SEM. (F) Formation of MitoNeoOH in the heart in response to superoxide; 25 nmol MitoNeoD and 2.5 nmol MitoPQ were given via tail vein injection. After 60 min, hearts were removed and processed as in (D), enabling MitoNeo and MitoNeoOH levels to be assessed by LC-MS/MS. Data are means ± SEM from three to six mice. *p < 0.05 by Student's t test. See also Figure S7.

It should be possible to use MitoNeoD to assess mitochondrial O_2_^⋅−^ formation *in vivo* by LC-MS/MS, relative to deuterated internal standards, as was done for H_2_O_2_ by use of the mitochondria-targeted mass spectrometric probe MitoB (Cochemé et al., 2011). To develop the LC-MS/MS assay we first established the fragmentation of MitoNeoOH and MitoNeo, as well as their deuterated internal standards (Figure 7A). This led to a sensitive LC-MS/MS assay for these two molecules (Figures S7A and S7B). The formation of MitoNeoOH from MitoNeoD when exposed to O_2_^⋅−^ was then quantified by LC-MS/MS, which showed that there was a steady accumulation of MitoNeoOH that was abolished by adding SOD, and decreased by bubbling with argon (Figure 7B). Therefore we have established an LC-MS/MS assay that responds to O_2_^⋅−^.

To extend MitoNeoD to the *in vivo* assessment of O_2_^⋅−^, it is important to normalize the formation of MitoNeoOH to the levels of its precursor *in vivo* (Cochemé et al., 2011). This was not possible for MitoNeoOH as the LC-MS/MS analysis of its precursor MitoNeoD gave inconsistent results due to variable oxidation upon extraction and analysis. To overcome this, while still being able to normalize MitoNeoOH formation, we treated the sample with the hydride acceptor, chloranil (Zielonka et al., 2006), which oxidized MitoNeoD to MitoNeo (Figures S7C–S7E) while leaving MitoNeoOH unchanged (Figure S7F). Then we measured the amount of MitoNeoOH and MitoNeo: the amount of MitoNeo thus measured corresponds to the sum of MitoNeoD and MitoNeo in the sample at the time of extraction. From this we could determine relative O_2_^⋅−^ formation within a tissue from the amount of MitoNeoOH formed relative to sum of MitoNeo and MitoNeoOH measured after reaction with chloranil. We call this sum Σ MitoNeo_×_ as it is the total amount of all MitoNeo variants (MitoNeoD, MitoNeo, and MitoNeoOH) in the tissue at extraction (Figure 7C). To assess this *in vitro* we exposed MitoNeoD to O_2_^⋅−^ and assessed the formation of MitoNeoOH over time by measuring MitoNeoOH/Σ MitoNeo_×_ (Figure 7D). This showed an increase in MitoNeoOH/Σ MitoNeo_×_ in the presence of O_2_^⋅−^ that was blocked by degrading O_2_^⋅−^ by SOD (Figure 7D). Therefore, the assessment of the MitoNeoOH/Σ MitoNeo_×_ can be used to assess changes in O_2_^⋅−^ *in vitro*.

### Assessment of Mitochondrial O_2_^⋅−^ Production *In Vivo* Using MitoNeoD

To see if MitoNeoD can assess mitochondrial O_2_^⋅−^ formation *in vivo* we focused on the mouse heart, because mitochondrial ROS production has been implicated in a multitude of cardiac pathologies (Chouchani et al., 2016). The incorporation of the TPP cation into MitoNeoD should lead to its rapid accumulation within heart mitochondria following intravenous injection, as has been shown for other TPP compounds (Porteous et al., 2010). We assessed this by injecting MitoNeoD into mice intravenously by a tail vein and then measured MitoNeoOH and MitoNeo in the heart and expressed this as Σ MitoNeo_×_ over time (Figure 7E). This showed a very rapid uptake of MitoNeoD into the heart within a few minutes of injection that was then gradually lost over time, with a half-life of ∼1 hr, consistent with other TPP compounds (Porteous et al., 2010). Interestingly, MitoNeoOH was a small proportion of Σ MitoNeo_×_ in the heart, consistent with low levels of mitochondrial O_2_^⋅−^ production in the normoxic heart and the relative stability of MitoNeoD *in vivo* (Figure 7E). To see if MitoNeoOH levels responded to an increase in mitochondrial O_2_^⋅−^ production *in vivo*, we administered MitoNeoD to mice at the same time as MitoPQ, which selectively induces mitochondrial O_2_^⋅−^ production in the heart (Robb et al., 2015). MitoPQ increased the MitoNeoOH/Σ MitoNeo_×_ ratio markedly in the hearts (Figure 7F). Therefore, MitoNeoD can be used to assess mitochondrial O_2_^⋅−^ production *in vivo*.

## Discussion

Mitochondrial O_2_^⋅−^ production plays a central role in pathology and redox signaling. However, progress in understanding these aspects of O_2_^⋅−^ physiology has been challenging due to the difficulties in measuring O_2_^⋅−^. While O_2_^⋅−^ assessments work well in simple systems, in cells O_2_^⋅−^ measurement by changes in fluorescence of probes such as HE or MitoSOX Red lacks selectivity due to non-specific oxidation by other ROS to E^+^ or MitoSOX (Zielonka and Kalyanaraman, 2010). Furthermore, fluorescence is greatly enhanced by intercalation into DNA, potentially distorting fluorescent signaling. As determined by the Kalyanaraman laboratory, the formation of the 2-hydroxy forms of E^+^, or of MitoSOX, are specific for O_2_^⋅−^; however, to assess these 2-hydroxy derivatives requires analysis by HPLC. Finally, the measurement of O_2_^⋅−^ *in vivo* is a particular challenge.

To address these issues, here we have developed an approach that can be used to interrogate mitochondrial O_2_^⋅−^ production in mitochondria, cells, and *in vivo* models, using fluorescence, RP-HPLC, and LC-MS/MS. By incorporating neopentyl groups we prevented the intercalation of MitoNeo and MitoNeoOH into DNA, hence their fluorescence is unaffected by interactions with DNA. Furthermore, the use of a C-D bond at a crucial point in the structure of MitoNeoD enhanced its stability against background oxidation and increased its selectivity for O_2_^⋅−^. The use of MitoNeoD to assess O_2_^⋅−^ production can also be extended to *in vivo* situations by the application of LC-MS/MS to the analysis. This will enable the role of mitochondrial O_2_^⋅−^ metabolism to be assessed *in vivo*, which is a major unmet need in the field. While here we have focused on using MitoNeoD to assess mitochondrial O_2_^⋅−^, future work will develop NeoD to measure O_2_^⋅−^ elsewhere in the cell.

The development of MitoNeoD is potentially of great use. Even so, limitations exist. In applying MitoNeoD to assess O_2_^⋅−^ *in vivo* by LC-MS/MS, a constraint is that each time point requires destructive tissue extraction *ex vivo*. A further point is that the initial radical cation intermediate generated by the oxidation of MitoNeoD can potentially be formed by other one-electron oxidants as well as O_2_^⋅−^ (Figure S1) (Michalski et al., 2014, Robinson et al., 2006, Zielonka and Kalyanaraman, 2010). The selectivity of the generation of MitoNeoOH for O_2_^⋅−^ still remains; however, in theory the formation of MitoNeoOH could be increased by an elevation in other one-electron oxidants without a change in O_2_^⋅−^ itself. Of course, these limitations also apply to the use of MitoSOX Red and HE. While fluorescence measurement of the oxidation of MitoNeoD will still be affected by the parallel formation of both MitoNeo and MitoNeoOH, the separation of excitation/emission wavelengths and the greater selectivity of MitoNeoD for O_2_^⋅−^ over other oxidants means that fluorescence changes in MitoNeoD/NeoD are a more reliable (but not absolute) indicator of changes in O_2_^⋅−^ than HE/MitoSOX Red. Furthermore, the lack of DNA intercalation of MitoNeo/Neo compounds prevents the dramatic enhancement of E^+^/MitoSOX fluorescence that may be susceptible to distortion, for example by changes in amount or accessibility of nuclear or mtDNA.

In summary, we have developed a versatile and robust set of methodologies to assess changes in mitochondrial O_2_^⋅−^ from isolated mitochondria to animal models *in vivo*. This development should help us better understand the many roles of mitochondrial O_2_^⋅−^ production in health and disease.

## Significance

**The measurement of O**_**2**_^**⋅−**^ **is critically important for many aspects of biology. However, current approaches are artifact prone and not applicable *in vivo*. Here we have developed a new probe, MitoNeoD, which is accumulated selectively by mitochondria and there reacts with O**_**2**_^**⋅−**^ **itself to generate a selective product, MitoNeoOH. This can then be assessed by fluorescence or by RP-HPLC to assess O**_**2**_^**⋅−**^ **in isolated mitochondria and in cells. More significantly, the assessment of MitoNeoOH formation from MitoNeoD can also be done *in vivo*, which has not been possible previously. This approach will enable the role of mitochondrial O**_**2**_^**⋅−**^ **formation *in vivo* to be analyzed and its role in pathology and cell signaling determined.**

## STAR★Methods

### Key Resources Table

**Table:** 

REAGENT or RESOURCE	SOURCE	IDENTIFIER
**Chemicals, Peptides, and Recombinant Proteins**		

Neo, *d*_*5*_*-*Neo	This paper	N/A
NeoH/D	This paper	N/A
NeoOH, *d*_*5*_*-*NeoOH	This paper	N/A
MitoNeo, *d*_*15*_*-*MitoNeo	This paper	N/A
MitoNeoH/D	This paper	N/A
MitoNeoOH, *d*_*15*_*-*MitoNeoOH	This paper	N/A
See chemistry procedures for the synthesis of intermediate compounds	This paper	N/A
Ethidium bromide (E^+^)	Sigma-Aldrich	Cat#09-0617SAJ-100mL; CAS: 1239-45-8
MitoSOX Red	Thermo Fisher Scientific	Cat#M36008
NaBH_4_	Sigma-Aldrich	Cat#452882-100G; CAS: 16940-66-2
NaBD_4_	Sigma-Aldrich	Cat#205591-1G; CAS: 15681-89-7
Fremy’s salt	Sigma-Aldrich	Cat#220930-1G; CAS: 14293-70-0
Xanthine oxidase	Sigma-Aldrich	Cat#X4500-5UN; CAS: 9002-17-9
Hypoxanthine	Sigma-Aldrich	Cat#H9377-5G; CAS: 68-94-0
Ferricytochrome *c*	Sigma-Aldrich	Cat#C2037
Superoxide dismutase	Sigma-Aldrich	Cat#S8160
Catalase	Sigma-Aldrich	Cat#C3515
Rotenone	Sigma-Aldrich	Cat#R8875-1G; CAS: 83-79-4
Succinate	Sigma-Aldrich	Cat#224731-5G; CAS: 150-90-3
FCCP	Sigma-Aldrich	Cat#C2920-10MG; CAS: 370-86-5
Chloranil	Sigma-Aldrich	Cat#232017-25G; CAS: 118-75-2
MitoPQ	Abcam	Cat#ab146819; CAS:146819-28-8
Menadione	Sigma-Aldrich	Cat#M5625-25G; CAS: 58-27-5
λ DNA HindIII Digest	NEB	Cat#N3012S
φ X174 DNA-HaeIII Digest	NEB	Cat#N3026S
Salmon sperm DNA	Sigma-Aldrich	Cat#D1626-1G; CAS: 438545-06-3
zirconium oxide beads	Next Advance	Cat#ZROb05
0.9-2.0 mm diameter stainless steel beads	Next Advance	Cat#SSBI4b

**Experimental Models: Cell Lines**		

Human: Flp-In™ T-Rex^TM^ HEK293T	Invitrogen	Cat#R&8007
Human: HeLa	ATCC	Cat#ATTC CCL-2
Mouse: C2C12	ATCC	Cat# ATTC C3H

**Experimental Models: Organisms/Strains**		

Mouse: C57BL/6J	Charles River Laboratories	664
Rat: Wistar	Charles River Laboratories	003

**Software and Algorithms**		

Masslynx		
NIS-Elements	Waters	http://www.waters.com/waters/en_US/MassLynx-MS-Software/nav.htm?locale=en_US&cid=513662
Chimera	Nikon	http://www.nikonmetrology.com/en_EU/Products/Software/Imaging-Software/NIS-Elements-Microscope-Imaging-Software
VMD	UCSF Resource for Biocomputing, Visualization, and Informatics	https://www.cgl.ucsf.edu/chimera/
ChromQuest	NIH Center for Biomolecular Modeling and Bioinformatics	http://www.ks.uiuc.edu/Research/vmd/
GraphPad Prism	Thermo Fisher Scientific	https://www.thermofisher.com/order/catalog/product/INQSOF012
GeneSnap	GraphPad Software	https://www.graphpad.com/scientific-software/prism/
Other	SynGene	http://www.syngene.com/genesnap

### Contact for Reagent and Resource Sharing

Further information and requests for resources and reagents should be directed to and will be fulfilled by the Lead Contact Dr Michael Murphy (mpm@mrc-mbu.cam.ac.uk) or by the co-corresponding author Prof Richard Hartley (Richard.Hartley@glasgow.ac.uk).

### Experimental Model and Subject Details

#### Cell Lines

Mouse C2C12 and human HeLa cells were obtained from American Type Culture Collection (ATCC). Human HEK293T Flp-In™ T-Rex™ were obtained from Invitrogen. All cell lines were cultured in DMEM medium supplemented with 10% fetal bovine serum (FBS), 100 U/ml penicillin and 100 μg/ml streptomycin, at 37°C in an atmosphere of 5% CO_2_ and 100% humidity.

#### Mice and Rats

All the procedures were carried out in accordance with the UK Animals (Scientific Procedures) Act 1986 and the University of Cambridge Animal Welfare Policy. Male C57BL/6 mice were obtained from Charles Rivers Laboratories (Margate, UK). Female Wistar rats were obtained from the Charles River Laboratories (Margate, UK)). Mice and rats were maintained in specific pathogen-free facilities with *ad lib* food and water until 8-12 and 10-12 weeks of age, respectively. Animals were killed by stunning and cervical dislocation.

### Method Details

#### Chemicals

All reagents were purchased from commercial sources, unless otherwise stated. Neo, NeoOH, MitoNeo, MitoNeoOH and their deuterated analogues (*d*_*5*_-Neo, *d*_*5*_-NeoOH, *d*_*15*_-MitoNeo and *d*_*15*_-MitoNeoOH) were synthesized as is summarised in Figures 1C and S2A. Full experimental details and structural assignment are included below. Stock solutions (10 mM) in absolute ethanol were stored at -20°C with negligible decomposition observed over months. Stock solutions of MitoNeoH/D were prepared as follows: ∼100 μL MitoNeo (10 mM in EtOH) was placed in a 15 mL Falcon tube, H_2_O and dichloromethane (CH_2_Cl_2_; ∼200 μL of each) were added, the tube flushed with argon, then ∼5 mg NaBH_4_/NaBD_4_ (Sigma-Aldrich) were added, the tube closed, followed by vortexing (5 s). The reaction was easily monitored by the change in color from deep purple (MitoNeo/Neo) to pale green (MitoNeoH/D). Then the lower organic layer was quickly removed to an argon-flushed Eppendorf, the residual aqueous layer was further extracted (∼100 μL CH_2_Cl_2_) and the organic layers combined and evaporated under argon. The residue was weighed and dissolved in EtOH to make up a 10 mM stock, aliquots of which were evaporated under argon to generate a pale-green solid that was stored at -20°C until use. Then single aliquots were dissolved in absolute EtOH to obtain a ∼10 mM stock solution that was flushed with argon and stored on ice, shielded from light and discarded after use. NeoH/D was synthesized from Neo and treated in the same way as MitoNeoH/D. MitoSOX was prepared from MitoSOX Red (Thermo Fisher Scientific) by oxidation in air.

#### Optical Measurements

UV-visible absorption was measured using a Shimadzu UV-2501PC spectrophotometer with a thermostatted cuvette holder. Fluorescence spectra were collected at RT in EtOH, in KCl buffer (120 mM KCl, 10 mM HEPES, 1 mM EGTA, pH 7.2 (KOH)) (MitoNeo series) or KCl buffer supplemented with 20% (v/v) EtOH in a 1 mL cuvette (Neo series) using a Shimadzu RF-5301PC spectrofluorophotometer (Shimadzu Scientific Instruments Inc., Japan). The slit widths were 3 and 5 nm for excitation and emission light, respectively. 3D spectra were collected with excitation and emission light wavelength range 400-780 nm (excitation) and 500-780 (emission) with 20 nm increment and 1 nm sampling interval. Fluorescence time course measurements were performed in a 3 mL cuvette at 37 **°**C and the excitation and emission wavelengths were 544 and 605 nm respectively (MitoNeoOH) and 548 and 599 nm respectively (NeoOH).

#### O_2_^⋅-^ Generation

The generation of O_2_^⋅-^ was done using 5 mU/mL xanthine oxidase (XO, Sigma-Aldrich), 1 mM hypoxanthine (HX, Sigma-Aldrich) in KCl buffer (pH 7.2) at 37°C. Sustained O_2_^⋅-^ generation was confirmed by the SOD-sensitive reduction of 20 μM ferricytochrome *c* (Sigma-Aldrich) at 550 nm (ɛ_red–ox_ = 21 mM^-1^cm^-1^), which showed that these conditions reduced ferricytochrome *c* at an initial rate of 0.6 mM/min.

#### Procedure for Docking Ethidium and Neo into DNA

The modelling was done with the program Chimera v. 1.10.2 (Resource for Biocomputing, Visualization, and Informatics at the University of California, San Francisco, http://www.cgl.ucsf.edu/chimera/) (Pettersen et al., 2004), using the Amber ff14SB force field (Maier et al., 2015) for the nucleic acids and GAFF (Wang et al., 2004) with AM1-BCC charges for the intercalators. Images were produced with VMD v. 1.9.2 (NIH Center for Biomolecular Modeling and Bioinformatics, http://www.ks.uiuc.edu/Research/vmd/) (Humphrey et al., 1996). In brief, the modelling procedure applied may be described as manual rigid docking. The modelling was based on the X-ray structure (PDB ID 452D) (Todd et al., 1999) of a 6-bp piece of double-stranded DNA (d(CGTACG)) with a molecule of DACA intercalating between each of the terminal d(CG) pairs (DACA = *N*-(2-dimethylamino)ethyl)acridine-4-carboxamide). In the crystal, the duplexes form chains, with another molecule of DACA bound in-between the d(CG) pairs of adjacent oligomers. This structure was used as no structure of an oligonucleotide duplex with intercalated ethidium (E^+^) is available; however, the binding modes of E^+^ and DACA are very similar (a structure of E^+^ bound to a CG duplex is available (Jain and Sobell, 1984). All non-nucleic acid components of the structure, including DACA, were removed. A pre-optimised molecule of E^+^ was placed manually in the same position as previously occupied by an intercalating DACA, with the substituents on the central ring sticking out into the major groove. Two orientations of E^+^ were considered, obtained by flipping the molecule by 180°. The structures were optimised, keeping the nucleic acid scaffold fixed. Neo was docked in the same way, considering again two orientations as well as a rotamer about the C^ar^–N bond that alleviates the worst clash between the neopentyl group and the backbone.

#### Pulse Radiolysis

The rate constants for the reaction of O_2_^⋅-^ with both NeoH and MitoNeoH (Reaction 6) were determined by following the formation of their one-electron oxidised radical spectra in real time upon pulse radiolysis (3 Gy in 200 ns), using the University of Auckland facility (Anderson et al., 1997). Radical spectra are presented as the changes in absorption per Gy. Due to the poor water solubility of the compounds, experiments were conducted in water:ethanol solutions (50:50) saturated with air:N_2_O gas (50:50). Under basic conditions (pH 11) the exclusively formed α-hydroxyethylperoxyl radical (CH_3_CH(OO^**.**^)OH) quickly breaks down (< 1 μs) upon reaction with OH^**-**^ (*k*_5_ = 4 × 10^9^ M^-1^ s^-1^) to form O_2_^⋅-^ (Bothe et al., 1983) and the rate constant for its reaction with increasing concentrations of the substrates was monitored at 475 nm. Phosphate buffer, which is also known to speed the breakdown of the α-hydroxyethylperoxyl radical to O_2_^⋅-^ (Bothe et al., 1983), could not be used as it induced precipitation of the compounds. The reaction of the α-hydroxyethylperoxyl radical with the compounds (Reaction 7) was studied in solutions of natural pH (ca. 6.0).

(Equation 1) H_2_O ˆˆˆˆˆ→
e^-^_aq_,
^**.**^OH,
H^**.**^,
H_2_O_2_,
H_2_,
H_3_O^**+**^

(Equation 2) N_2_O +
e^**-**^_aq_
→ ^**.**^OH +
OH^**-**^ +
N_2_

(Equation 3) ^**.**^OH/H^**.**^ +
CH_3_CH_2_OH →
H_2_O/H_2_ +
CH_3_CH(^**.**^)OH

(Equation 4) CH_3_CH(^**.**^)OH +
O_2_ →
CH_3_CH(OO^**.**^)OH

(Equation 5) CH_3_CH(OO^**.**^)OH +
OH^**-**^ →
CH_3_CHO +
O_2_^**.-**^ +
H_2_O

(Equation 6) O_2_^**.-**^ +
NeoH/MitoNeoH + H_2_O →
NeoH^**+.**^/MitoNeoH^**+.**^ +
HO_2_^**-**^ +
OH^**-**^

(Equation 7) CH_3_CH(OO^**.**^)OH +
NeoH/MitoNeoH →
NeoH^**+.**^/MitoNeoH^**+.**^ +
CH_3_CH(OO^**-**^)OH

(Equation 8) NeoH^**+.**^/MitoNeoH^**+.**^ +
H_2_O ⇋
NeoH(-H^+^)/MitoNeoH(-H^+^) +
H_3_O^+^

#### Isolation of Rat Liver Mitochondria

Female Wistar rats (Charles River Laboratories) liver mitochondria were isolated by homogenisation and differential centrifugation in STE buffer (250 mM sucrose, 5 mM Tris, 1 mM EDTA, pH 7.4 (HCl)) at 4 **°**C and stored on ice until use. Protein concentration was determined by the biuret assay using bovine serum albumin (BSA) as a standard. Mitochondrial incubations at 2 mg protein/mL were at 37°C in KCl buffer (120 mM KCl, 10 mM HEPES, 1 mM EGTA. pH 7.2 (KOH)) unless stated otherwise.

#### Ion-selective Electrode Measurements

The uptake of MitoNeoH, MitoNeo and MitoNeoOH by energized mitochondria was measured using an ion-selective electrode sensitive to the TPP cation. The electrode was constructed as described previously and the voltage was measured relative to an Ag/AgCl reference electrode (World Precision Instruments) (Asin-Cayuela et al., 2004, Kamo et al., 1979). The electrodes were connected to a PowerLab data acquisition system via a front-end pH amplifier and the output was recorded with Chart v. 4.2 software (ADInstruments, https://www.adinstruments.com/products/labchart). Mitochondria (2 mg protein/mL) were incubated at 37 **°**C in a stirred thermostatted chamber containing 3 mL KCl buffer and 4 μg/mL rotenone (Sigma-Aldrich). The electrode response was calibrated by five sequential injections of 1 μM MitoNeoH. Mitochondria were energized with succinate (10 mM) (Sigma-Aldrich) and uncoupled by addition of 500 nM FCCP (Sigma-Aldrich).

#### Reverse Phase HPLC Analysis

Samples were dissolved in 1 or 1.5 mL 25 % Buffer B (0.1% (v/v) trifluoroacetic acid (TFA) in acetonitrile (ACN))/75% Buffer A (0.1% (v/v) TFA in H_2_O) and filtered (0.22 μm PVDF filter (Millipore)). Samples were then loaded *via* a 2 mL injection loop onto a C18 RP-HPLC column (Jupiter 300 Å, Phenomenex) with a Widepore C18 guard column. Samples were eluted at 2 mL/min using a Gilson 321 pump to generate the following gradient: 5-55% Buffer B 0-4 min; 55-70% Buffer B 4-16 min; 70-100% Buffer B 16-18 min; 100% Buffer B 18-21 min, 100-5% Buffer B 21-23 min. A_220_ of column eluent was measured using a Gilson UV/Vis 151 spectrophotometer and fluorescence (550 nm excitation, 590 nm emission for MitoNeoOH: note these wavelengths are different from those used in aqueous buffer) was measured using a Shimadzu RF-10A_XL_ fluorescence detector (λ_excitation_ = 328 nm; λ_emission_ = 375 nm) connected in series with the RP-HPLC system described above. Outputs were monitored using Chart v. 4.2 software (ADInstruments, https://www.adinstruments.com/products/labchart).

#### Mouse Experiments

Male C57BL/6 mice were administered MitoNeoD (25 nmol) with or without MitoPQ (2.5 nmol) as a 100 μL bolus in 0.9% saline by tail vein injection and killed at various times subsequently. Hearts were then isolated and frozen in liquid nitrogen. The concentration of MitoNeoD used (25 nmol/mouse) is lower than the amounts routinely used for iv injection of other TPP compounds such as MitoQ, which show no toxic effects on mice. There was no observed toxicity in the control mice exposed to MitoNeoD compared to saline injected controls.

#### LC-MS/MS Analysis

To analyse MitoNeo compounds in tissues, 50 ± 5 mg wet weight tissue was placed in a 2 mL Eppendorf tubes and to this was added 248 μL KCl buffer, and 10 μM internal standards (ISs; 5 μM *d*_*15*_-MitoNeoOH/10 μM *d*_*15*_-MitoNeo) and 2 μL chloranil (Sigma-Aldrich) (10 mM in acetone). A spatula was used to add a volume of beads ∼equivalent to that of the tissue sample (for liver these were 0.5 mm diameter zirconium oxide beads and for heart they were 0.9-2.0 mm diameter stainless steel beads, both from Next Advance). The tissue was then homogenized in a Bullet Blender (Storm 24(BBY24M) Next Advance) for 3 min at speed 10 and then incubated at 37°C for 30 min rotating at 1000 rpm (lids closed). For extraction of non-tissue samples the homogenisation step was omitted. The homogenate was then supplemented with 1 mL butan-2-ol/methanol (3:1) and sonicated in a water bath (Branson 3800, Bransonic Ultrasonic Bath, CPX from Emerson Industrial Automation) at RT for 1 h. The samples were then centrifuged for 10 min at 16,000 x g and the supernatant was transferred to a fresh 2 mL tube and dried in a Speed Vac under vacuum at 40°C. To the dried residue was added 400 μL 40% HPLC grade methanol/0.1% formic acid (FA)/60% HPLC grade water. This was vortexed for 15 min, centrifuged at 16,000 x g for 10 min, the supernatant was vacuum filtered and 300 μL was transferred to mass spectrometry vials and stored at 4°C until analysis.

Samples were analysed by LC-MS/MS using an I-class Acquity LC attached to a Xevo TQ-S triple quadrupole mass spectrometer (Waters), analysed using MassLynx software (Waters, http://www.waters.com/waters/en_US/MassLynx-MS-Software/nav.htm?locale=en_US&cid=513662). Samples and standards in autosampler vials were placed in a refrigerated holder (4°C) while awaiting introduction by the autosampler. LC was performed at 30°C using an Acquity UPLC BEH C18 1.7 μm, 1 × 50 mm (Waters). The mobile phase consisted of 5% acetonitrile (ACN)/0.1% FA in water (buffer A) and 90% ACN/0.1% FA (buffer B) delivered as a linear gradient: 0-0.3 min, 5% B; 0.3-8 min, 5-100% B; 8-9 min, 100% B; 9-9.1 min, 100-5% B; 9.1-10 min, 5% B. The flow rate was 200 μL/min and the 2 μL sample volume was introduced via a flow-through needle. An in-line divert valve was used to divert eluent away from the mass spectrometer from 0-3 min and 7-10 min of the acquisition time. Multiple reaction monitoring (MRM) in positive ion mode was used to detect the compounds. The instrument parameters were: source spray voltage, 2.7 kV; ion source temperature, 150°C; cone voltage and collision energy were optimised for each compound. Nitrogen was used as the curtain gas and argon as the collision gas. For all experiments, a standard curve was prepared and processed in parallel using the appropriate biological material or buffer spiked with *d*_*15*_-MitoNeo and *d*_*15*_-MitoNeoOH ISs and a range of MitoNeo or MitoNeoOH amounts. Standard curves for the response of MitoNeo and MitoNeoOH relative to its deuterated IS against concentration were linear over the range 1-1,000 pmol with R^2^ routinely > 0.99.

#### Analysis of Cell Uptake of MitoNeo Probes by Confocal Microscopy

To assess uptake of MitoNeo and MitoNeoOH into mitochondria within cells, C2C12 cells were seeded at 75,000 cells on 35 mm diameter glass bottom dish (Ibidi) and allowed to adhere overnight. The medium was removed by aspiration and replaced with Optimem, 10% FBS and 1% Glutamax (Invitrogen), containing the different probes ± 0.5 μM FCCP (Sigma-Aldrich). Cells were then placed on a temperature controlled (37 °C) chamber of an inverted microscope (Nikon A1R+) and visualized using a 63X objective lens (Nikon), 561 nm laser line for excitation and a spectral detector with galvano scanner for acquisition. Images were captured every min and analyzed using the NIS-Elements software (Nikon, http://www.nikonmetrology.com/en_EU/Products/Software/Imaging-Software/NIS-Elements-Microscope-Imaging-Software).

To assess the localisation of MitoNeo and MitoNeoOH by confocal microscopy we used the Flp-In T-Rex™ HEK293T cell line, which allows for the generation of stable doxycycline-inducible expression of transgenes by FLP recombinase-mediated integration. Cells were transfected at ∼50% confluence with the vectors pOG44 and pcDNA5/FRT/TO containing sequence of the genes to be expressed (mitochodrial-tagged GFP) with slight modification as previously described (Rorbach et al., 2012). 24 h after transfection the selective antibiotics hygromycin (100 μg/mL, Invitrogen) and blasticidin (15 μg/mL, Invitrogen) were added with selective media. The day before the experiment cells were grown to 50% confluence on 35 mm diameter glass bottom dish (Ibidi) and induced with 10 mg/mL doxycyclin (Invitrogen) for 24 h to allow the expression of the mitochondria-targeted GFP. On the day of experiment, cells were incubated with MitoNeo or MitoNeoOH (5 μM) for 10 min at 37 °C and then visualized on a temperature controlled (37 °C) chamber of a Nikon A1R+ inverted confocal microscope using a 63X objective lens (Nikon), 488 and 561 nm laser lines for excitation and a spectral detector with galvano scanner for acquisition. Images were analyzed using the NIS-Elements software (Nikon). Identical settings were used in comparing images to analyse changes in intensity due to ROS production.

#### Agarose Gel Electrophoresis

DNA (10 μg of a mixture of a Lambda DNA HindIII digest (NEB) and a PhiX 174 RF DNA HaeIII digest (NEb)) was mixed with the indicated concentrations of compound in DNA loading buffer (2.5% Ficoll®-400, 11 mM EDTA, 3.3 mM Tris-HCl, 0.017% SDS, 0.015% bromophenol blue, pH 8.0). The DNA was then resolved on a 0.9% (w/v) agarose gel in TBE buffer (89 mM Tris, 89 mM borate, 2 mM EDTA. pH 8.0 (HCl)) for 15-20 min at 400 mA/100 V. The gel was then visualized on a UV transilluminator and photographed with GeneSnap software (SynGene, http://www.syngene.com/genesnap).

#### MTS Cell Proliferation Assay

C2C12 cells were seeded in a 96 well plate at 10,000 cells/well, grown overnight and then various concentrations of MitoNeo, MitoNeoH and MitoNeoOH were added and compared with no additions or ethanol carrier. Menadione (50 M; Md) was used as a positive control for cell death. After incubation for 17.5 h cell viability was assessed by the MTS assay and the absorbance measured at 490 nm. Data are means ± SD for 8 wells.

#### Chemical Syntheses

##### Synthesis of MitoNeo, Neo and Their Derivatives

The synthesis of MitoNeoH (Figure 1C) began from commercially available 3,8-diaminophenanthridine **1**, which underwent double reductive amination with pivalaldehyde in the presence of sodium triacetoxyborohydride and trifluoroacetic acid (TFA) to give the bis(neopentyl) derivative **2** in quantitative yield. Selective *N-*alkylation of the phenanthridine nitrogen atom could be achieved using freshly prepared 6-bromohexyl triflate **3** to give *N-*(bromohexyl)phenanthridinium salt **4**. Phenanthridines are generally poor nucleophiles so the use of an alkyl triflate is preferred (Lee and Shin, 2005, Ross et al., 2000). Fortunately, the neopentyl groups block reactivity on the 3- and 8-amino groups but do not reduce the electron-donating ability of the amino groups, unlike the carbamate derivatives of 3,8-diaminophenanthridine that are generally used to make *N-*alkyl phenanthridinum salts. The latter, was partially purified by chromatography, and then reacted with triphenylphosphine in toluene under reflux to give MitoNeo in moderate yield after HPLC purification and ion exchange. *d*_*15*_-MitoNeo was prepared from *N-*(bromohexyl)phenanthridinium salt **4** in the same way using *d*_*15*_-triphenylphosphine. Reduction of MitoNeo in a two-phase water-dichloromethane mixture under argon by NaBH_4_/NaBD_4_ gave MitoNeoH/D. MitoNeoOH was prepared by reaction of MitoNeo with Fremy’s salt (potassium nitrosodisulfonate) (Zielonka et al., 2008) and was isolated as a red mesylate salt in modest yield after HPLC and ion exchange. *d*_*15*_-MitoNeoOH was prepared in the same way. A deuterated form of the dimer of MitoNeo was also prepared to serve as an IS for potential dimer formation (Kalyanaraman et al., 2014). NeoH and NeoOH were prepared in a similar way to MitoNeo (Figure S2A). Ethylation of the bis(neopentyl) derivative **2** was achieved with ethyl triflate **5** to give Neo as the mesylate salt in good yield after ion exchange. *d*_*5*_-Neo was prepared in the same way using *d*_*5*_-ethyl triflate **6**. Reduction of Neo in a two-phase water-diethyl ether mixture under argon by NaBH_4_/NaBD_4_ gave NeoH/D. The regioselectivity of hydroxylation at C-2 to give MitoNeoOH or NeoOH was confirmed by ^1^H NMR, as H-7 is shielded by the ring current of the 6-phenyl group and remains a doublet in MitoNeoOH with a chemical shift similar to that of H-7 in MitoNeo (5.56 and 6.09 ppm respectively). The same is true for H-7 in NeoOH and Neo (5.91 and 5.81, respectively).

##### General

All reactions under an inert atmosphere were carried out using oven-dried or flame-dried glassware and solvents were added via syringe. Reagents were obtained from commercial suppliers and used without further purification. Dry solvents were collected from a Puresolv solvent purification system, obtained from commercial suppliers or dried in the laboratory. Ethanol was distilled from Mg turnings activated with iodine. ^1^H NMR spectra were obtained using Bruker-Avance III spectrometers operating at 500 and 400 MHz, ^13^C NMR spectra at 126 and 101 MHz respectively. Signal splitting patterns were described as: singlet (s), doublet (d), triplet (t), quartet (q), multiplet (m), broad singlet (s, broad), or any combination of the above. All coupling constants were recorded in Hz. DEPT was used to assign the signals in ^13^C NMR spectra as C, CH, CH_2_ and CH_3_. 2D techniques including COSY, HMBC and HSQC were used to aid assignment. All spectra were assigned using the following reference solvent peaks for residual non-deuterated solvent in the ^1^H NMR spectra and for the deuterated solvent in the ^13^C NMR spectra: CDCl_3_ (7.26 ppm for ^1^H NMR; 77.16 ppm for ^13^C NMR) CD_2_Cl_2_ (5.32 ppm for ^1^H NMR). ^1^H NMR analysis of the reaction product of NeoD with O_2_^⋅−^ was obtained by exposing NeoD (100 μM) to O_2_^⋅−^ by incubation with XO (0.5 U/mL) and hypoxanthine (1 mM) for 3 h at 37°C in 1:0.5:3.5 mixture of EtOH:PBS:H_2_O and then extracted into CHCl_3_, purified by HPLC (see method for NeoOH purification) and the ^1^H NMR spectrum obtained as above. HRMS (ESI^+^) spectra were collected on a Bruker MicroTOF-Q. IR spectra were obtained using Shimadzu FTIR-8400S. *R*_*f*_ values for phosphonium salts are concentration dependent and are reported as the maximum observed *R*_*f*_. Purification of products was carried out by recrystallization, column chromatography using silica gel (70-230 mesh) and reverse phase HPLC. Reverse phase HPLC buffers were 0.1% FA in H_2_O (Buffer A) and 100% ACN (Buffer B). C18 column (Phenomenex Gemini-NX 10μ C18 250 × 21.20 mm) was used. Samples dissolved in 3-5 mL of 25% ACN in 0.1% FA_(aq)_ were filtered manually through a 0.45 μm PTFE filter (Sartorius Stedim Biotech) and loaded onto a column via a 10 mL injection loop. Samples were eluted at 12 mL/min using different gradients (SpectraSystem P2000). A_220_ of the column eluent was detected using UV/Vis spectrophotometer (SpectroMonitor 3200) and visualized with ChromQuest software.

##### 3,8-Bis(neopentylamino)-6-phenylphenanthridine **2**

3,8-Diamino-6-phenylphenanthridine **1** (650 mg, 2.28 mmol, 1.00 eq.) and NaBH(OAc)_3_ (1.45 g, 6.83 mmol, 3.00 eq.) were mixed in TFA (0.81 mL, 11 mmol, 4.7 eq.). The mixture was cooled to −15°C under argon with an IPA/CO_2(s)_ bath. CH_2_Cl_2_ (6.5 mL) was added and the mixture stirred. 2,2-Dimethylpropanal (0.54 mL, 5.0 mmol, 2.2 eq.) was then added by syringe and the mixture allowed to stir for 2 h, then the mixture was partitioned between CH_2_Cl_2_ and H_2_O, then the aqueous extracted with CH_2_Cl_2_. The combined organic layers were washed with brine, then extracted with CH_2_Cl_2_ and the organics combined and solvent removed under reduced pressure. The material was dissolved in Et_2_O and precipitated with excess ethereal HCl (2 M), then allowed to settle overnight. The solid was filtered, washed with Et_2_O and dissolved in CH_2_Cl_2_ then washed with NaHCO_3(aq)_. The aqueous was re-extracted with CH_2_Cl_2_, then the combined organics dried over anhydrous MgSO_4_, filtered and the solvent removed under reduced pressure to give phenanthridine **2** as a foam (1.08 g, 99%). δ_H_ (CDCl_3_, 500 MHz): 8.28 (1H, d, *J* = 9.0 Hz, H-10), 8.19 (1H, d, *J =* 8.9 Hz, H-1), 7.68-7.63 (2H, m, H-2”, H-6”), 7.53-7.43 (3H, m, H-3”, H-4”, H-5”), 7.26 (1H, d, *J* = 2.3 Hz, H-4), 7.16 (1H, dd, *J* = 8.9, 2.5 Hz, H-9), 7.02-6.97 (2H, m, H-7, H-2), 3.91 (2H, apparent s, broad, 3-NHR, 8-NHR), 3.02 (2H, s, 8-NHC**H**_**2**_), 2.87 (2H, s, 3-NHC**H**_**2**_), 1.00 [9H, s, 8-NHCH_2_C(CH_3_)_3_)], 0.96 [9H, s, 3-NHCH_2_C(CH_3_)_3_)]. δ_C_ (126 MHz, CDCl_3_) 159.91 (C), 148.65 (C), 146.73 (C), 143.19 (C), 139.57 (C), 129.51 (CH), 128.56 (CH), 128.25 (CH), 126.56 (C), 125.12 (C), 122.36 (CH), 121.94 (CH), 120.51 (CH), 117.04 (CH), 116.17 (C), 107.43 (CH), 107.33 (CH), 55.88 (CH_2_), 55.84 (CH_2_), 32.04 (C), 31.97 (C), 27.58 (CH_3_), 27.56 (CH_3_). IR (ATR cm^-1^): 3424 (N-H), 3285 (N-H), 2953 (C-H), 2864 (C-H), 1620 (C=N), 1568 (C_Ar_=C_Ar_), 1512 (C_Ar_=C_Ar_). MS (EI^+^): 425 (M^+⋅^, 72%), 368 [M^+⋅^ –^.^C(CH_3_)_3_, 100]. HRMS: 425.2837. C_29_H_35_N_3_ requires requires M^+⋅^, 425.2831.

##### 6-Bromohexyl Trifluoromethanesulfonate **3**

1-Bromohexan-6-ol (0.72 mL, 5.5 mmol, 1.0 eq.) was added to a stirring solution of anhydrous CH_2_Cl_2_ (5 mL), anhydrous pyridine (0.40 mL, 5.0 mmol, 0.9 eq.) and triflic anhydride (1.0 mL, 6.1 mmol, 1.1 eq.) at 0°C under argon. The mixture was allowed to stir at 0°C for 1.5 h and quenched into CH_2_Cl_2_/H_2_O (50 mL). The layers were separated and organics were washed with H_2_O (2 × 30 mL), dried over anhydrous MgSO_4_, filtered and concentrated under reduced pressure to give triflate **3** as a pure yellow-brown oil (1.53 g, 88%). δ_H_ (400 MHz, CDCl_3_): 4.55 (2H, t, *J* = 5.9 Hz, C**H**_**2**_OTf), 3.40 (2H, t, *J* = 6.9 Hz, C**H**_**2**_Br), 1.91-1.80 (4H, m, **CH**_**2**_CH_2_OTf, **CH**_**2**_CH_2_Br), 1.55-1.40 (4H, m, RC**H**_**2**_C**H**_**2**_R’). δ_C_ (101 MHz, CDCl_3_): 118.6 (q, *J* = 320.6 Hz, CF_3_), 77.62 (CH_2_), 33.40 (CH_2_), 32.29 (CH_2_), 29.01 (CH_2_), 27.28 (CH_2_), 24.22 (CH_2_). ^1^H and ^13^C NMR data agree with literature.(Armstrong-Chong et al., 2004)

##### 3,8-Bis(neopentylamino)-5-(6’-bromohexyl)-6-phenylphenanthridinium Triflate **4**

Phenanthridine **2** (1.00 g, 2.35 mmol, 1.0 eq.) was added to a stirring solution of triflate **3** (750 mg, 2.40 mmol, 1.0 eq.) in anhydrous Et_2_O (12.5 mL) at –30°C under argon. The mixture was allowed to warm to RT and stirred over 16 h. The reaction was quenched into H_2_O (50 mL), extracted with CH_2_Cl_2_ (50 mL), the organic layer separated and dried over anhydrous MgSO_4_, filtered and concentrated under reduced pressure to give a purple foam. Column chromatography [SiO_2_, gradient from CH_2_Cl_2_-NEt_3_-ACN (91.5:1:7.5) to (83:2:15)] yielded triflate **4** as a purple glassy foam (990 mg, 52% approx. 85% pure). R_f_ [SiO_2_, CH_2_Cl_2_-NEt_3_-ACN (91.5:1:7.5)]: 0.22. δ_H_ (400 MHz, CDCl_3_): 8.30 (1H, d, *J* = 9.3 Hz, H-10), 8.15 (1H, d, *J* = 9.3 Hz, H-1), 7.82-7.73 (3H, m, Ph). 7.55 (1H, dd, *J* = 9.0, 2.2 Hz, H-9), 7.47 (1H, apparent s, broad, H-4), 7.43-7.38 (3H, m, Ph, H-2), 6.30 (1H, t, *J* = 5.9 Hz, 3-N**H**R), 6.04 (1H, d, *J* = 2.3 Hz, H-7), 4.83 (1H, t, *J* = 6.1 Hz, 8-N**H**R), 4.63 (2H, m, NC**H**_**2**_), 3.35 (2H, t, *J* = 6.6 Hz, C**H**_**2**_Br), 3.14 (2H, d, *J* = 5.8 Hz, 3-NHC**H**_**2**_tBu), 2.67 (2H, d, *J* = 5.6 Hz, 8-NHC**H**_**2**_tBu), 1.96-1.85 (2H, m, CH_2_-2’), 1.81-1.73 (2H, m, CH_2_-5’), 1.39-1.27 (4H, m, CH_2_-3’, CH_2_-4’), 1.09 (9H, s, 3-NHCH_2_**tBu**), 0.89 (9H, s, 8-NHCH_2_**tBu**). δ_C_ (101 MHz, CDCl_3_): 157.03 (C), 152.14 (C), 147.90 (C), 134.97 (C), 132.08 (C), 131.14 (CH), 129.69 (CH), 128.81 (C), 128.67 (CH), 128.19 (CH), 124.71 (C), 124.13 (CH), 121.89 (CH), 118.20 (CH), 117.47 (C), 104.32 (CH), 97.42 (CH), 55.04 (CH_2_), 54.79 (CH_2_), 53.42 (CH_2_), 33.78 (CH_2_), 32.78 (C), 32.47 (C), 32.16 (CH_2_), 28.88 (CH_2_), 27.66 (CH_3_), 27.56 (CH_3_), 27.22 (CH_2_), 25.36 (CH_2_). ν_Max_(ATR)cm^-1^: 3362 (N-H), 2949 (C-H), 2933 (C-H), 2862 (C-H), 1618 (C=N). MS (ESI^+^): 588 [M^+^ (^79^Br, phenanthridinium cation), 100%] and 590 [M^+^ (^81^Br, phenanthridinium cation), 100%]. HRMS: 588.2926 and 590.2913. C_35_H_47_^79^BrN_3_^+^ requires M^+^, 588.2948 and C_35_H_47_^81^BrN_3_^+^ requires M^+^, 590.2928.

##### 3,8-Bis(neopentylamino)-5-(6’-triphenylphosphoniohexyl)-6-phenylphenanthridinium Mesylate **(MitoNeo, Bis-mesylate Salt)**

6’-Bromohexylphenanthridinium triflate **4** (200 mg, 0.27 mmol, 1.0 eq.) was combined with triphenylphosphine (360 mg, 1.37 mmol, 5.0 eq.), the mixture was dried by azeotrope with anhydrous toluene (2.0 mL). This was repeated one more time and after removal of the solvent under reduced pressure anhydrous toluene (2.5 mL) was added to the mixture. The resulting solution was stirred for 24 h at reflux under argon. The mixture was allowed to cool to RT, the toluene removed with a pipette, the residue washed with toluene, dissolved in CHCl_3_ and concentrated under reduced pressure to give a purple solid. The crude mixture was purified using preparative HPLC method by separate injections of 15 mg crude for each purification. Gradient elution of buffers A and B was from 75:25 to 60:40 over 40 min and from 60:40 to 55:45 over the next 20 min. Pure fractions were collected and combined. Brine was added (addition of brine helps to extract aqueous layer containing ACN), the solution was extracted with CHCl_3_, dried over anhydrous MgSO_4_, filtered and concentrated under reduced pressure to give MitoNeo as a chloride salt. The solid was ion exchanged to the mesylate form [IRA 401 resin in mesylate form, loaded and eluted in MeOH-H_2_O (1:1)], furnishing mesylate MitoNeo as a purple glass (120 mg, 46%). t_R_ = 29 min. δ_H_ (500 MHz, CDCl_3_): 8.38 (1H, d, *J* = 9.3 Hz, H-1), 8.26 (1H, d, *J* = 9.3 Hz, H-10), 7.99 (1H, d, *J* = 2.1 Hz, H-4), 7.81-7.60 (19H, m, C-6-Ph, P**Ph**_**3**_, H-9), 7.39-7.29 (3H, m, C-6-Ph, H-2), 6.09 (1H, d, *J* = 2.4 Hz, H-7), 4.70 (2H, apparent s, broad, C**H**_**2**_-1’), 3.58-3.42 (2H, m, C**H**_**2**_-6’), 3.10 (2H, s, 3-NHC**H**_**2**_^t^Bu), 2.74 (6H, s, 2C**H**_**3**_SO_3_^-^), 2.67 (2H, s, 8-NHC**H**_**2**_^t^Bu), 1.85 (2H, apparent s, broad, C**H**_**2**_-2’), 1.55 (6H, apparent s, broad, C**H**_**2**_-3’, C**H**_**2**_-4’, C**H**_**2**_-5’), 1.03 (9H, s, 3-NHCH_2_^**t**^**Bu**), 0.86 (9H, s, 8-NHCH_2_^**t**^**Bu**). δ_C_ (126 MHz, CDCl_3_): 157.23 (C), 152.67 (C), 148.30 (C), 135.06 (d, *J* = 2.9 Hz, CH), 134.95 (C), 133.67 (d, *J* = 10.0 Hz, CH), 132.30 (C), 131.06 (CH), 130.54 (d, *J* = 12.5 Hz, CH), 129.72 (CH), 129.28 (CH), 128.89 (C), 128.09 (CH), 124.71 (C), 124.29 (CH), 121.87 (CH), 118.53 (d, *J* = 85.9 Hz, C), 117.24 (C), 116.23 (CH), 104.30 (CH), 99.73 (CH), 55.26 (CH_2_), 54.92 (CH_2_), 53.43 (CH_2_), 39.73 (CH_3_), 32.88 (C), 32.67 (C), 29.22 (CH_2_), 29.09 (CH_2_), 27.80 (CH_3_), 27.73 (CH_3_), 24.88 (CH_2_), 21.94 (d, *J* = 4.3 Hz, CH_2_), 21.38 (d, *J* = 51.0 Hz, CH_2_). HRMS (ESI^+^, m/z): found 385.7327. C_53_H_62_N_3_P (M^2+^, phenanthridinium dication) requires 385.7335. ^1^H and ^13^C NMR and HRMS data agree (Cairns et al., 2014).

##### 3,8-Bis(neopentylamino)-5-[6’-tri(pentadeuterophenyl)phosphoniohexyl]-6-phenylphenanthridinium Mesylate **(d**_**15**_**-MitoNeo, Bis-mesylate Salt)**

6’-Bromohexylphenanthridinium triflate **4** (146 mg, 0.20 mmol, 1.0 eq.) was combined with tri(pentadeuterophenyl)phosphine (277 mg, 0.99 mmol, 5.0 eq.), the mixture was dried by azeotrope with anhydrous toluene (2.0 mL). This was repeated one more time and after removal of the solvent under reduced pressure anhydrous toluene (2.0 mL) was added to the mixture. The resulting solution was stirred for 24 h at reflux under argon. The mixture was allowed to cool to RT, the toluene removed with a pipette, the residue washed with toluene, dissolved in CHCl_3_ and concentrated under reduced pressure to give a purple solid. The crude solid was purified using preparative HPLC method by separate injections of 15 mg crude for each purification. Gradient elution of buffers A and B was from 75:25 to 60:40 over 40 min and from 60:40 to 55:45 over the next 20 min. Pure fractions were collected and combined. Brine was added, the solution was extracted with CHCl_3_, dried over anhydrous MgSO_4_, filtered and concentrated under reduced pressure to give *d*_*15*_-MitoNeo as a chloride salt. The solid was ion exchanged to the mesylate form [IRA 401 resin in mesylate form, loaded and eluted in MeOH-H_2_O (1:1)], furnishing mesylate *d*_*15*_-MitoNeo as a purple glass (92 mg, 47%). t_R_ = 29 min. δ_H_ (500 MHz, CDCl_3_): 8.32 (1H, d, *J* = 9.3 Hz, H-1), 8.19 (1H, d, *J* = 9.2 Hz, H-10), 7.94 (1H, s, H-4), 7.71-7.63 (4H, m, C-6-Ph, H-9), 7.33-7.27 (3H, m, C-6-Ph, H-2), 7.06 (1H, apparent s, broad, 3-N**H**R), 5.95 (1H, d, *J* = 2.4 Hz, H-7), 5.23 (1H, apparent s, broad, 8-N**H**R), 4.76-4.58 (2H, m, C**H**_**2**_-1’), 3.59-3.45 (2H, m, C**H**_**2**_-6’), 3.07 (2H, s, 3-NHC**H**_**2**_^t^Bu), 2.69 (6H, s, 2C**H**_**3**_SO_3_^-^), 2.62 (2H, s, 8-NHC**H**_**2**_^t^Bu), 1.83 (2H, apparent s, broad, C**H**_**2**_-2’), 1.62-1.45 (6H, m, C**H**_**2**_-3’, C**H**_**2**_-4’, C**H**_**2**_-5’), 1.01 (9H, s, 3-NHCH_2_^**t**^**Bu**), 0.82 (9H, s, 8-NHCH_2_^**t**^**Bu**). δ_C_ (126 MHz, CDCl_3_): 157.33 (C), 152.72 (C), 148.23 (C), 135.00 (C), 134.98-134.30 (m, CD), 133.72-132.83 (m, CD), 132.30 (C), 131.10 (CH), 130.35-129.90 (m, CD), 129.75 (CH), 129.13 (CH), 128.95 (C), 128.11 (CH), 124.73 (C), 124.31 (CH), 121.94 (CH), 118.35 (d, *J* = 85.7 Hz, C), 117.24 (C), 116.20 (CH), 104.51 (CH), 99.86 (CH), 55.28 (CH_2_), 54.96 (CH_2_), 53.48 (CH_2_), 39.74 (CH_3_), 32.91 (C), 32.66 (C), 29.26 (CH_2_), 29.11 (CH_2_), 27.82 (CH_3_), 27.74 (CH_3_), 24.91 (CH_2_), 21.94 (d, *J* = 4.7 Hz, CH_2_), 21.39 (d, *J* = 51.4 Hz, CH_2_). IR ν_max_(cm^-1^): 3352 (N-H), 3240 (N-H), 3056 (C_Ar_-H), 2951 (C-H), 2866 (C-H), 1619 (C=N), 1476 (C_Ar_=C_Ar_). HRMS (ESI^+^, m/z): found 393.2787. C_53_H_47_D_15_N_3_P (M^2+^, phenanthridinium dication) requires 393.2806.

##### 3,8-Bis(neopentylamino)-5-(6’-triphenylphosphoniohexyl)-6-phenylphenanthridine **(MitoNeoH)** and 3,8-Bis(neopentylamino)-5-(6’-triphenylphosphoniohexyl)-6-deutero-6-phenylphenanthridine **(MitoNeoD)**

**MitoNeo** (bis-mesylate salt, 30 mg, 0.03 mmol, 1.0 eq.) was added to a tube flushed with argon and filled with 3.0 mL of degassed H_2_O, 3.0 mL of degassed CH_2_Cl_2_ and shaken to dissolve under argon. NaBH_4_ (12 mg, 0.32 mmol, 10 eq.) was added afterwards and the mixture was shaken for 5 min under argon in the dark and the phases were left to separate for 1 min. The organic layer was syringed out to a vial flushed with argon; the aqueous layer was extracted again with 3.0 mL of degassed CH_2_Cl_2_ and the organic layer was syringed out to a vial. The organics were combined and the solvent was evaporated by argon flux furnishing **MitoNeoH** as a pale green foam (24 mg, 99%). The crude was used without purification in the next step. An identical result was obtained when MitoNeo was reduced with NaBD_4_ to give MitoNeoD. UV-visible and HPLC data are given in the main text. MitoNeoH/D are air-sensitive so NMR data were obtained in a separate experiment in which 4 mg of MitoNeo was dissolved in 0.6 mL of CD_2_Cl_2_, placed under argon in an NMR tube and a solution of ∼1 mg of NaBH_4_/NaBD_4_ in 0.7 mL of D_2_O was added, the tube was shaken until the purple colour disappeared and the NMR spectra were obtained. The two distinct spins systems in MitoNeo-H were assigned by COSY while the H1-H2-H4 spin system was assigned to the more upfield protons. Note: H_2_O and CH_2_Cl_2_ (10 mL each) should be degassed separately in flasks with Suba-Seals® by bubbling the contents of a large argon-filled balloon through a long needle with a vent present (10-15 min) and kept under argon. All reaction vessels (tubes, vials) should be kept under positive argon pressure; all the needles and syringes should be flushed with argon before use. All the manipulations with a reduced product should be done in the dark.

###### MitoNeoH

δ_H_ (400 MHz, CD_2_Cl_2_): 7.91-7.76 (3H, m, PPh_3_), 7.76-7.63 (6H, m, PPh_3_), 7.65-7.51 (6H, m, PPh_3_), 7.36 (1H, d, *J* = 8.3 Hz, H-1), 7.33 (1H, d, *J* = 8.3 Hz, H-10), 7.18-7.12 (5H, m, C-6-Ph), 6.50 (1H, dd, *J* = 8.4, 2.3 Hz, H-9), 6.37 (1H, d, *J* = 2.4 Hz, H-7), 6.09 (1H, dd, *J* = 8.2, 2.2 Hz, H-2), 5.95 (1H, d, *J* = 2.2 Hz, H-4), 5.23 (1H, s, H-6), 3.51-3.37 (1H, m, N*CH*_*A*_CH_B_), 3.12-2.94 (3H, m, NCH_A_*CH*_*B*_, PCH_2_), 2.88 (2H, s, NCH_2_), 2.83 (2H, s, NCH_2_), 1.70-1.58 (6H, m, 3 × CH_2_), 1.44-1.34 (2H, m, CH_2_), 0.98 (9H, s, NHCH_2_^**t**^**Bu**)**,** 0.96 (9H, s, NHCH_2_^**t**^**Bu**)**.**

δ_C_ (101 MHz, CD_2_Cl_2_): 149.95 (C), 147.87 (C), 144.56 (C), 143.95 (C), 135.88 (d, *J* = 3.1 Hz, CH), 135.77 (C), 133.95 (d, *J* = 9.9 Hz, CH), 131.12 (d, *J* = 12.5 Hz, CH), 128.89 (CH), 127.72 (CH), 127.06 (CH), 123.57 (CH), 122.83 (CH), 121.39 (C), 118.35 (d, *J* = 86.3 Hz, C), 113.62 (C), 113.12 (CH), 110.70 (CH), 102.64 (CH), 97.61 (CH), 66.92 (CH), 56.44 (CH_2_), 56.37 (CH_2_), 49.69 (CH_2_), 32.29 (C), 32.21 (C), 30.61 (d, *J* = 15.6 Hz, CH_2_), 28.01 (CH_3_), 27.93 (CH_3_), 27.18 (CH_2_), 26.84 (CH_2_), 22.95 (d, *J* = 51.5 Hz, CH_2_), 22.75 (d, *J* = 4.7 Hz, CH_2_).

###### MitoNeoD

δ_H_ (400 MHz, CD_2_Cl_2_): 7.88-7.80 (3H, m, PPh_3_), 7.72-7.65 (6H, m, PPh_3_), 7.60-7.53 (6H, m, PPh_3_), 7.36 (1H, broad d, *J* = 8.3 Hz, H-1), 7.33 (1H, broad d, *J* = 8.7 Hz, H-10), 7.17-7.11 (5H, m, C-6-Ph), 6.49 (1H, broad d, *J* = 8.7 Hz, H-9), 6.37 (1H, broad s, H-7), 6.09 (1H, broad d, *J* = 8.3 Hz, H-2), 5.95 (1H, broad s, H-4), 3.43 (1H, dd, *J* = 13.9, 7.0 Hz, N*CH*_*A*_CH_B_), 3.11-2.92 (3H, m, NCH_A_*CH*_*B*_, PCH_2_), 2.88 (2H, s, NCH_2_), 2.83 (2H, s, NCH_2_), 1.70-1.58 (6H, m, 3 × CH_2_), 1.44-1.34 (2H, m, CH_2_), 0.98 (9H, s, NHCH_2_^**t**^**Bu**)**,** 0.96 (9H, s, NHCH_2_^**t**^**Bu**)**.**

##### 3,8-Bis(neopentylamino)-5-(6’-tri(pentadeuterophenyl)phosphoniohexyl)-6-phenylphenanthridine **(d**_**15**_**-MitoNeoH)**

***d***_***15***_**-MitoNeo** (bis-mesylate salt, 30 mg, 0.03 mmol, 1.0 eq.) was added to a tube flushed with argon and filled with 3.0 mL of degassed H_2_O, 3.0 mL of degassed CH_2_Cl_2_ and shaken to dissolve under argon. NaBH_4_ (12 mg, 0.31 mmol, 10 eq.) was added afterwards and the mixture was shaken for 5 min under argon in the dark and the phases were left to separate for 1 min. The organic layer was syringed out to a vial flushed with argon; the aqueous layer was extracted again with 3.0 mL of degassed CH_2_Cl_2_ and the organic layer was syringed out to a vial. The organics were combined and the solvent was evaporated by argon flux furnishing **d**_**15**_**-MitoNeoH** as a pale green foam (24 mg, 99%). The crude was used without purification in the next step. N.B. The same precautions apply as for **MitoNeoH**.

***d***_***15***_**-**MitoNeoH/D are air-sensitive so NMR data were obtained in a separate experiment in which 8 mg ***d***_***15***_**-**MitoNeo was dissolved in 0.6 mL of CD_2_Cl_2_, placed under argon in an NMR tube and a solution of ∼1.2 mg of NaBH_4_/NaBD_4_ in 0.7 mL of D_2_O was added, the tube was shaken until the purple colour disappeared and the NMR spectra were obtained. Similarly, ***d***_***15***_**-**MitoNeoD was synthesised from 15 mg ***d***_***15***_**-**MitoNeo and 2.4 mg NaBD_4_. The two distinct spins systems in MitoNeo-H were assigned by COSY while the H1-H2-H4 spin system was assigned to the more upfield protons. Note: H_2_O and CH_2_Cl_2_ (10 mL each) should be degassed separately in flasks with Suba-Seals® by bubbling the contents of a large argon-filled balloon through a long needle with a vent present (10-15 min) and kept under argon. All reaction vessels (tubes, vials) should be kept under positive argon pressure; all the needles and syringes should be flushed with argon before use. All the manipulations with a reduced product should be done in the dark.

###### *d*_*15*_-MitoNeoH

δ_H_ (400 MHz, CD_2_Cl_2_): 7.36 (1H, d, *J* = 8.3 Hz, H-1), 7.33 (1H, d, *J* = 8.5 Hz, H-10), 7.17-7.12 (5H, m, Ph), 6.50 (1H, dd, *J* = 8.5, 2.4 Hz, H-9), 6.37 (1H, d, *J* = 2.5 Hz, H-7), 6.09 (1H, dd, *J* = 8.3, 2.1 Hz, H-2), 5.94 (1H, d, *J* = 2.3 Hz, H-4), 5.23 (1H, s, H-6), 3.48-3.39 (1H, m, N*CH*_*A*_CH_B_), 3.08-3.01 (1H, m, NCH_A_*CH*_*B*_, PCH_2_), 3.00-2.93 (2H, m, PCH_2_), 2.88 (2H, s, NCH_2_), 2.83 (2H, s, NCH_2_), 1.70-1.46 (6H, m, 3 × CH_2_), 1.43-1.34 (2H, m, CH_2_), 0.98 (9H, s, NHCH_2_^**t**^**Bu**)**,** 0.96 (9H, s, NHCH_2_^**t**^**Bu**). δ_C_ (101 MHz, CD_2_Cl_2_): 149.93 (C), 147.83 (C), 144.55 (C), 143.93 (C), 135.76 (C), 135.73-135.10 (m, CD), 133.83-133.25 (m, CD), 130.96-130.26 (m, CD), 128.88 (CH), 127.72 (CH), 127.04 (CH), 123.55 (CH), 122.82 (CH), 121.36 (C), 118.10 (d, *J* = 86.1 Hz, C), 113.6 (C), 113.10 (CH), 110.69 (CH), 102.62 (CH), 97.58 (CH), 66.91 (CH), 56.42 (CH_2_), 56.35 (CH_2_), 49.67 (CH_2_), 32.27 (C), 32.20 (C), 30.65 (d, *J* = 15.6 Hz, CH_2_), 27.99 (CH_3_), 27.92 (CH_3_), 27.17 (CH_2_), 26.82 (CH_2_), 22.95 (d, *J* = 51.5 Hz, CH_2_), 22.75 (d, *J* = 4.7 Hz, CH_2_).

###### *d*_*15*_-MitoNeoD

δ_H_ (400 MHz, CD_2_Cl_2_): 7.36 (1H, d, *J* = 8.3 Hz, H-1), 7.33 (1H, d, *J* = 8.5 Hz, H-10), 7.17-7.11 (5H, m, Ph), 6.50 (1H, dd, *J* = 8.5, 2.5 Hz, H-9), 6.37 (1H, d, *J* = 2.5 Hz, H-7), 6.09 (1H, dd, *J* = 8.3, 2.2 Hz, H-2), 5.95 (1H, d, *J* = 2.2 Hz, H-4), 3.43 (1H, dd, *J* = 13.4, 6.2 Hz, N*CH*_*A*_CH_B_), 3.10-2.92 (3H, m, NCH_A_*CH*_*B*_, PCH_2_), 2.88 (2H, s, NCH_2_), 2.83 (2H, s, NCH_2_), 1.70-1.48 (6H, m, 3 × CH_2_), 1.44-1.33 (2H, m, CH_2_), 0.98 (9H, s, NHCH_2_^**t**^**Bu**)**,** 0.96 (9H, s, NHCH_2_^**t**^**Bu**). δ_C_ (101 MHz, CD_2_Cl_2_): 149.93 (C), 147.83 (C), 144.55 (C), 143.88 (C), 135.70 (C), 135.66-135.03 (m, CD), 134.05-132.97 (m, CD), 131.14-130.14 (m, CD), 128.87 (CH), 127.70 (CH), 127.03 (CH), 123.53 (CH), 122.81 (CH), 121.35 (C), 118.11 (d, *J* = 86.1 Hz, C), 113.59 (C), 113.07 (CH), 110.63 (CH), 102.58 (CH), 97.57 (CH), 56.41 (CH_2_), 56.34 (CH_2_), 49.60 (CH_2_), 32.27 (C), 32.19 (C), 30.63 (d, *J* = 15.8 Hz, CH_2_), 27.98 (CH_3_), 27.91 (CH_3_), 27.18 (CH_2_), 26.81 (CH_2_), 22.90 (d, *J* = 51.5 Hz, CH_2_), 22.75 (d, *J* = 4.4 Hz, CH_2_). Note: C-6 C-D not resolved from baseline.

##### 3,8-Bis(neopentylamino)-2-hydroxy-5-(6’-triphenylphosphoniohexyl)-6-phenylphenanthridinium Mesylate **(MitoNeoOH, Bis-mesylate Salt)**

Fremy’s salt (27 mg, 0.10 mmol, 4.0 eq.) was dissolved in 20 mL of phosphate buffer (pH 7.4), through which argon was bubbled during 10 min prior to salt addition. Freshly prepared **MitoNeoH** (20 mg, 0.03 mmol, 1.0 eq.), which was used immediately after the synthesis, was dissolved in 0.5 mL of CH_2_Cl_2_, transferred to the buffer and the mixture was stirred at RT under argon for 1 h in the dark. The solution was extracted with CHCl_3_ (3 × 20 mL), the organic layers combined, dried over anhydrous MgSO_4_, filtered and concentrated under reduced pressure to give a raspberry red foam. The crude foam was purified using preparative HPLC method by separate injections of 10 mg crude for each purification. Gradient elution of buffers A and B was from 75:25 to 65:35 over 40 min and from 65:35 to 58:42 over the next 20 min. Pure fractions were collected and combined. Brine was added, the solution was extracted with CHCl_3_, dried over anhydrous MgSO_4_, filtered and concentrated under reduced pressure to give **MitoNeoOH** as a chloride salt. The solid was ion exchanged to the mesylate form [IRA 401 resin in mesylate form, loaded and eluted in MeOH-H_2_O (1:1)], furnishing **mesylate MitoNeoOH** as a raspberry red glass (7 mg, 28%). t_R_ = 28 min. δ_H_ (500 MHz, CDCl_3_): 11.76 (1H, s, O**H**), 8.52 (1H, d, *J* = 9.3 Hz, H-10), 8.44 (1H, s, H-1), 7.88-7.63 (18H, m, P**Ph**_**3**_, C-6-Ph), 7.52 (1H, dd, *J* = 9.1, 2.3 Hz, H-9), 7.24-7.16 (2H, m, C-6-Ph), 6.65 (1H, s, H-4), 5.86 (1H, d, *J* = 2.3 Hz, H-7), 5.27 (1H, t, *J* = 5.1 Hz, 3-N**H**R), 4.90 (1H, apparent s, broad, 8-N**H**R), 4.49 (2H, apparent s, broad, C**H**_**2**_-1’), 3.75-3.49 (2H, m, C**H**_**2**_-6’), 3.05 (2H, d, *J* = 5.1 Hz, 3-NHC**H**_**2**_^t^Bu), 2.75 (6H, s, 2C**H**_**3**_SO_3_^-^), 2.63 (2H, s, 8-NHC**H**_**2**_^t^Bu), 1.75 (2H, apparent s, broad, C**H**_**2**_-2’), 1.67-1.51 (4H, m, C**H**_**2**_-3’, C**H**_**2**_-5’), 1.49-1.33 (2H, m, C**H**_**2**_-4’), 0.99 (9H, s, 3-NHCH_2_^**t**^**Bu**), 0.84 (9H, s, 8-NHCH_2_^**t**^**Bu**). HRMS (ESI^+^, m/z): found 393.7299. C_53_H_62_N_3_OP (M^2+^, phenanthridinium dication) requires 393.7310.

##### 3,8-bis(neopentylamino)-2-hydroxy-5-(6’-tri(pentadeuterophenyl)phosphoniohexyl)-6-phenylphenanthridinium Mesylate **(d**_**15**_**-MitoNeoOH, Bis-mesylate Salt)**

Fremy’s salt (27 mg, 0.10 mmol, 4.0 eq.) was dissolved in 20 mL of phosphate buffer (pH 7.4), through which argon was bubbled during 10 min prior to salt addition. Freshly prepared ***d***_***15***_**-MitoNeoH** (20 mg, 0.03 mmol, 1.0 eq.), which was used immediately after the synthesis, was dissolved in 0.5 mL of CH_2_Cl_2_, transferred to the buffer and the mixture was stirred at RT under argon for 1 h in the dark. The solution was extracted with CHCl_3_ (3 × 20 mL), the organic layers combined, dried over MgSO_4_, filtered and concentrated under reduced pressure to give a raspberry red foam. The crude mixture was purified using preparative HPLC method by separate injections of 10 mg crude for each purification. Gradient elution of buffers A and B was from 75:25 to 65:35 over 40 min and from 65:35 to 58:42 over the next 20 min. Pure fractions were collected and combined. Brine was added, the solution was extracted with CHCl_3_, dried over anhydrous MgSO_4_, filtered and concentrated under reduced pressure to give ***d***_***15***_**-MitoNeoOH** as a chloride salt. The solid was ion exchanged to the mesylate form [IRA 401 resin in mesylate form, loaded and eluted in MeOH-H_2_O (1:1)], furnishing **mesylate *d***_***15***_**-MitoNeoOH** as a raspberry red glass (7 mg, 28%). t_R_ = 28 min. δ_H_ (500 MHz, CDCl_3_): 11.81 (1H, s, O**H**), 8.52 (1H, d, *J* = 9.2 Hz, H-10), 8.47 (1H, s, H-1), 7.68-7.62 (3H, m, Ph), 7.52 (1H, dd, *J* = 9.3, 2.4 Hz, H-9), 7.24-7.17 (2H, m, Ph), 6.66 (1H, s, H-4), 5.87 (1H, d, *J* = 2.4 Hz, H-7), 5.30 (1H, apparent s, broad, 3-N**H**R), 4.88 (1H, apparent s, broad, 8-N**H**R), 4.48 (2H, apparent s, broad, C**H**_**2**_-1’), 3.71-3.60 (2H, m, C**H**_**2**_-6’), 3.05 (2H, d, *J* = 5.4 Hz, 3-NHC**H**_**2**_^t^Bu), 2.75 (6H, s, 2C**H**_**3**_SO_3_^-^), 2.64 (2H, d, *J* = 3.5 Hz, 8-NHC**H**_**2**_^t^Bu), 1.78 (2H, apparent s, broad, C**H**_**2**_-3’), 1.69-1.50 (4H, m, C**H**_**2**_-3’, C**H**_**2**_-5’), 1.45-1.35 (2H, m, C**H**_**2**_-4’), 0.99 (9H, s, 3-NHCH_2_^**t**^**Bu**), 0.84 (9H, s, 8-NHCH_2_^**t**^**Bu**). HRMS (ESI^+^, m/z): found 401.2762. C_53_H_47_D_15_N_3_OP (M^2+^, phenanthridinium dication) requires 401.2781.

##### Ethyl Trifluoromethanesulfonate **5**

Purchased from commercial supplier and used as supplied.

##### Pentadeuteroethyl Trifluoromethanesulfonate **6**

Triflic anhydride (3.60 mL, 21.1 mmol, 1.1 eq.) was dissolved in anhydrous CH_2_Cl_2_ (15 mL) and stirred at 0°C under argon for 5 min. Anhydrous pyridine (1.60 mL, 19.2 mmol, 1.0 eq.) and anhydrous pentadeuteroethanol (1.00 g, 19.2 mmol, 1.0 eq.) were dissolved in anhydrous CH_2_Cl_2_ (10 mL) and added dropwise to the solution of triflic anhydride. The mixture was stirred at 0°C under argon for 25 min. Then the solution was quickly washed with H_2_O (15 mL), dried over anhydrous MgSO_4_, filtered and 15-20 mL of solvent were evaporated under reduced pressure with no heating. The crude in residual organics (5-10 mL) was distilled to furnish **6** as a transparent liquid (1.10 g, 32%); bp 110-115°C. δ_C_ (126 MHz, CDCl_3_): 118.97 (q, *J* = 320.0 Hz, C), 74.40-73.25 (m, CD_2_), 15.13-13.72 (m, CD_3_). The compound is known in the literature but not characterized (Anderson and Harruna, 1987). Chemical shifts in ^13^C NMR are close to those reported for ethyl triflate (Shima et al., 1996).

##### 3,8-Bis(neopentylamino)-5-ethyl-6-phenylphenanthridinium Mesylate **(Neo Mesylate)**

Phenylphenanthridine **2** (400 mg, 0.94 mmol, 1.0 eq.) was dissolved in anhydrous CH_2_Cl_2_ (4.0 mL) and stirred at 0°C under argon. Ethyl triflate **5** (0.98 mL, 0.75 mmol, 0.8 eq.) was added followed by a further addition of CH_2_Cl_2_ (1.0 mL). The ice bath was removed and the solution was stirred for 15 h at RT under argon. The mixture was washed with H_2_O (10 mL), the organic layer separated, dried over anhydrous MgSO_4_, filtered and concentrated under reduced pressure to give a purple foam. The residue was loaded on a silica plug (∼50 g), washed with Et_2_O (8 × 100 mL), eluted with CHCl_3_ and the solvent was concentrated under reduced pressure. The residue was purified using preparative HPLC method by separate injections of 15 mg of crude for each purification. Gradient elution of buffers A and B was from 60:40 to 45:55 over 45 min. Pure fractions were collected and combined. Brine was added, the solution was extracted with CHCl_3_, dried over anhydrous MgSO_4_, filtered and concentrated under reduced pressure to give **Neo** as a chloride salt. The solid was ion exchanged to the mesylate form [IRA 401 resin in mesylate form, loaded and eluted in MeOH-H_2_O (1:1)], furnishing **Neo mesylate** as a purple foam (252 mg, 49%). t_R_ = 20 min.

δ_H_ (500 MHz, CDCl_3_): 8.03 (1H, d, *J* = 9.3 Hz, H-1), 7.77 (1H, d, *J* = 9.3 Hz, H-10), 7.70-7.59 (5H, m, Ph, H-9, H-4), 7.36 (1H, dd, *J* = 9.4, 2.0 Hz, H-2), 7.26-7.22 (2H, m, Ph), 7.02 (1H, t, *J* = 6.1 Hz, 3-N**H**R), 5.81 (1H, d, *J* = 2.4 Hz, H-7), 5.76 (1H, t, *J* = 6.2 Hz, 8-N**H**R), 4.68 (2H, q, *J* = 7.1 Hz, NC**H**_**2**_CH_3_), 3.07 (2H, d, *J* = 6.1 Hz, 3-NHC**H**_**2**_^t^Bu), 2.78 (3H, s, C**H**_**3**_SO_3_^-^), 2.57 (2H, d, *J* = 6.0 Hz, 8-NHC**H**_**2**_^t^Bu), 1.43 (3H, t, *J* = 7.1 Hz, NCH_2_C**H**_**3**_), 1.05 (9H, s, 3-NHCH_2_^**t**^**Bu**), 0.79 (9H, s, 8-NHCH_2_^**t**^**Bu**). δ_C_ (126 MHz, CDCl_3_): 156.32 (C), 152.38 (C), 148.23 (C), 134.48 (C), 132.18 (C), 130.77 (CH), 129.38 (CH), 129.30 (CH), 128.36 (C), 127.96 (CH), 124.40 (C), 123.66 (CH), 121.21 (CH), 117.82 (CH), 117.06 (C), 103.01 (CH), 97.95 (CH), 55.03 (CH_2_), 54.68 (CH_2_), 49.07 (CH_2_), 39.58 (CH_3_), 32.77 (C), 32.57 (C), 27.69 (CH_3_), 27.55 (CH_3_), 14.33 (CH_3_). HRMS (ESI^+^, m/z): found 454.3211. C_31_H_40_N_3_ ([M]^+^) requires 454.3217.

##### 3,8-Bis(neopentylamino)-5-(pentadeuteroethyl)-6-phenylphenanthridinium Mesylate **(d**_**5**_**-Neo Mesylate)**

Phenylphenanthridine **2** (100 mg, 0.24 mmol, 1.0 eq.) was dissolved in anhydrous CH_2_Cl_2_ (1.0 mL) and stirred at 0°C under argon. Pentadeuteroethyl triflate **6** (43 mg, 0.24 mmol, 1.0 eq.) was added followed by a further addition of CH_2_Cl_2_ (0.5 mL). The ice bath was removed and the solution was stirred for 20 h at RT under argon. H_2_O (3 mL) was added, the mixture was shaken and the layers separated. The organic layer was dried over anhydrous MgSO_4_, filtered and concentrated under reduced pressure to give a purple foam. The residue was loaded on a short silica plug, washed with Et_2_O (5 × 100 mL), eluted with CHCl_3_ and the solvent was concentrated under reduced pressure. The crude mixture was purified using preparative HPLC method by separate injections of 15 mg of crude for each purification. Gradient elution of buffers A and B was from 60:40 to 45:55 over 45 min. Pure fractions were collected and combined. Brine was added, the solution was extracted with CHCl_3_, dried over anhydrous MgSO_4_, filtered and concentrated under reduced pressure to give ***d***_***5***_**-Neo** as a chloride salt. The solid was ion exchanged to the mesylate form [IRA 401 resin in mesylate form, loaded and eluted in MeOH-H_2_O (1:1)], furnishing ***d***_***5***_**-Neo mesylate** as a purple foam (57 mg, 44%). t_R_ = 20 min. δ_H_ (500 MHz, CDCl_3_): 8.12 (1H, d, *J* = 9.3 Hz, H-1), 7.85 (1H, d, *J* = 9.3 Hz, H-10), 7.81 (1H, apparent s, broad, H-4), 7.75-7.62 (3H, m, Ph), 7.58 (1H, dd, *J* = 9.2, 2.5 Hz, H-9), 7.37 (1H, dd, *J* = 9.3, 2.1 Hz, H-2), 7.32-7.27 (2H, m, Ph), 7.12 (1H, t, *J* = 6.1 Hz, 3-N**H**R), 5.88 (1H, d, *J* = 2.4 Hz, H-7), 5.39 (1H, t, *J* = 6.1 Hz, 8-N**H**R), 3.11 (2H, d, *J* = 6.0 Hz, 3-NHC**H**_**2**_^t^Bu), 2.81 (3H, s, C**H**_**3**_SO_3_^-^), 2.62 (2H, d, *J* = 6.0 Hz, 8-NHC**H**_**2**_^t^Bu), 1.08 (9H, s, 3-NHCH_2_^**t**^**Bu**), 0.83 (9H, s, 8-NHCH_2_^**t**^**Bu**). δ_C_ (126 MHz, CDCl_3_): 156.50 (C), 152.65 (C), 147.92 (C), 134.66 (C), 132.27 (C), 131.84 (CH), 129.15 (CH), 129.00 (CH), 128.63 (C), 128.05 (CH), 124.34 (C), 123.72 (CH), 121.43 (CH), 117.38 (CH), 116.98 (C), 103.63 (CH), 97.82 (CH), 55.13 (CH_2_), 54.84 (CH_2_), 39.61 (CH_3_), 32.86 (C), 32.54 (C), 27.76 (CH_3_), 27.59 (CH_3_). HRMS (ESI^+^, m/z): found 459.3514. C_31_H_35_D_5_N_3_ ([M]^+^) requires 459.3531.

##### 3,8-Bis(neopentylamino)-5-ethyl-6-phenylhydrophenanthridine **(NeoH)** and 3,8-Bis(neopentylamino)-6-deutero-5-ethyl-6-phenylhydrophenanthridine **(NeoD)**

**Neo mesylate** (30 mg, 0.06 mmol, 1.0 eq.) was added to a tube flushed with argon and filled with degassed H_2_O (3.0 mL) and degassed Et_2_O (3.0 mL). The mixture was shaken to dissolve under argon. Then NaBH_4_ (21 mg, 0.55 mmol, 10 eq.) was added to the mixture, it was shaken for 5 min under argon in the dark and the phases were left to separate for 1 min. The organic layer was syringed out to a vial flushed with argon; the aqueous layer was extracted again with degassed Et_2_O (3.0 mL) and the organic layer was syringed out to a vial. The organics were combined and the solvent was evaporated by argon flux furnishing **NeoH** as a pale green foam (24 mg, 99%). The crude was characterized by ^1^H NMR and used without purification in the next step. An identical result was obtained when Neo was reduced with NaBD_4_ to give NeoD. UV-visible and HPLC data are given in Figures S3 and S4. NeoH/D are air-sensitive and NMR data were conveniently obtained for NeoD in a separate experiment in which 4.0 mg Neo was dissolved in 0.6 mL of CD_2_Cl_2_, placed under argon in an NMR tube and a solution of 1.0 mg of NaBH_4_/D_4_ in 0.7 mL of D_2_O was added, the tube was shaken until the purple colour disappeared and the NMR spectra were obtained. N.B. The same precautions apply as for **MitoNeoH**.

###### NeoH

δ_H_ (400 MHz, CD_2_Cl_2_): 7.44 (1H, d, *J* = 8.5 Hz, H-10), 7.42-7.40 (1H, m, H-1), 7.21-7.13 (5H, m, C-6-Ph), 6.55 (1H, dd, *J* = 8.6, 2.3 Hz, H-9), 6.33 (1H, d, *J* = 2.3 Hz, H-7), 6.09 (1H, d, *J* = 8.3, H-2), 5.97 (1H, s, H-4), 3.42 (1H, dq, *J* = 14.3, 7.0 Hz, N*CH*_*A*_CH_B_), 3.19 (1H, dq, *J* = 14.0, 7.0 Hz, NCH_A_*CH*_*B*_), 2.90 (2H, s, NCH_2_), 2.84 (2H, s, NCH_2_), 1.18 (2H, t, *J* = 7.0 Hz, CH_3_), 1.00 (9H, s, NHCH_2_^**t**^**Bu**)**,** 0.96 (9H, s, NHCH_2_^**t**^**Bu**).

Due to overlap of the C-H signal with the CD_2_Cl_2_ signal in the ^1^H NMR spectrum, the reduction of Neo with NaBH_4_/D_4_ (1.5 mg scale) was also obtained in CDCl_3._ Note: The reduction in CD_2_Cl_2_ is cleaner than that observed in CDCl_3_.

###### NeoH

δ_H_ (400 MHz, CDCl_3_): 7.47-7.32 (2H, m, H-1, H-10), 7.17-7.06 (5H, m, Ph), 6.47 (1H, d, *J* = 8.6 Hz, H-9), 6.22 (1H, d, *J* = 2.5 Hz, H-7), 6.04 (1H, d, *J* = 8.3 Hz, H-2), 5.90 (1H, s, H-4), 5.25 (1H, s, C-H), 3.40-3.27 (1H, m, N*CH*_*A*_CH_B_), 3.17-3.07 (1H, m, NCH_A_*CH*_*B*_), 2.84 (2H, s, NCH_2_), 2.75 (2H, s, NCH_2_), 1.16-1.04 (3H, m, CH_2_*CH*_*3*_), 0.93 (9H, s, NHCH_2_^**t**^**Bu**)**,** 0.89 (9H, s, NHCH_2_^**t**^**Bu**)**.**

###### NeoD

δ_H_ (400 MHz, CD_2_Cl_2_): 7.44 (1H, d, *J* = 8.4 Hz, H-10), 7.41 (1H, d, *J* = 8.4 Hz, H-1), 7.22-7.12 (5H, m, C-6-Ph), 6.55 (1H, dd, *J* = 8.4, Hz, H-9), 6.33 (1H, s, H-7), 6.09 (1H, d, *J* = 8.3, H-2), 5.97 (1H, s, H-4), 3.47-3.37 (1H, m, N*CH*_*A*_CH_B_), 3.23-3.13 (1H, m, NCH_A_*CH*_*B*_), 2.90 (2H, s, NCH_2_), 2.85 (2H, s, NCH_2_), 1.21-1.13 (3H, m, CH_3_), 1.00 (9H, s, NHCH_2_^**t**^**Bu**)**,** 0.96 (9H, s, NHCH_2_^**t**^**Bu**).

δ_H_ (400 MHz, CDCl_3_): 7.42-7.37 (2H, m, H-1, H-10), 7.17-7.05 (5H, m, C-6-Ph), 6.47 (1H, d, *J* = 8.4 Hz, H-9), 6.23 (1H, s, H-7), 6.04 (1H, d, *J* = 7.9 Hz, H-2), 5.90 (1H, s, H-4), 3.37-3.27 (1H, m, N*CH*_*A*_CH_B_), 3.15-3.07 (1H, m, NCH_A_*CH*_*B*_), 2.84 (2H, s, NCH_2_), 2.76 (2H, s, NCH_2_), 1.14-1.08 (3H, m, CH_2_*CH*_*3*_), 0.93 (9H, s, NHCH_2_^**t**^**Bu**)**,** 0.89 (9H, s, NHCH_2_^**t**^**Bu**).

##### 3,8-Bis(neopentylamino)-5-(pentadeuteroethyl)-6-phenylhydrophenanthridine **(d**_**5**_**-NeoH)**

***d***_***5***_**-Neo mesylate** (10 mg, 0.02 mmol, 1.0 eq.) was added to a tube flushed with argon and filled with 1.0 mL of degassed H_2_O, 1.0 mL of degassed Et_2_O and shaken to dissolve under argon. NaBH_4_ (7.0 mg, 0.18 mmol, 10 eq.) was added afterwards and the mixture was shaken for 5 min under argon in the dark and the phases were left to separate for 1 min. The organic layer was syringed out to a vial flushed with argon; the aqueous layer was extracted again with 1.0 mL of degassed Et_2_O and the organic layer was syringed out to a vial. The organics were combined and the solvent was evaporated by argon flux furnishing **d**_**5**_**-NeoH** as a pale green foam (8 mg, 99%). The crude was used without purification in the next step.

N.B. The same precautions apply as for **MitoNeoH**.

##### 3,8-Bis(neopentylamino)-5-ethyl-2-hydroxy-6-phenylphenanthridinium Mesylate **(NeoOH Mesylate)**

Fremy’s salt (43 mg, 0.18 mmol, 4.0 eq.) was dissolved in 20 mL of phosphate buffer (pH 7.4), through which argon was bubbled during 10 min before addition of salt. Freshly prepared **NeoH** (20 mg, 0.04 mmol, 1.0 eq.), which was used immediately after the synthesis, was dissolved in 0.5 mL of Et_2_O, transferred to the buffer and the mixture was stirred at RT under argon for 1 h in the dark. The solution was extracted with CHCl_3_ (3 × 20 mL), the organic layers combined, dried over anhydrous MgSO_4_, filtered and concentrated under reduced pressure to give a raspberry red foam. The crude mixture was purified using preparative HPLC method by separate injections of 10 mg crude for each purification. Gradient elution of buffers A and B was from 58:42 to 50:50 over 45 min. Pure fractions were collected and combined. Brine was added, the solution was extracted with CHCl_3_, dried over anhydrous MgSO_4_, filtered and concentrated under reduced pressure to give **NeoOH** as a chloride salt. The solid was ion exchanged to the mesylate form [IRA 401 resin in mesylate form, loaded and eluted in MeOH-H_2_O (1:1)], furnishing **mesylate NeoOH** as a raspberry red glass (10 mg, 48%). δ_H_ (500 MHz, CDCl_3_): 8.32 (1H, s, H-10), 7.96 (1H, apparent s, broad, H-1), 7.68-7.62 (3H, m, Ph), 7.40 (2H, m, Ph), 7.33 (1H, apparent s, broad, H-9), 6.68 (1H, s, H-4), 6.08 (1H, apparent s, broad, 3-NHR), 5.91 (1H, d, *J* = 1.9 Hz, H-7), 4.57 (2H, q, *J* = 7.2 Hz, NC**H**_**2**_CH_3_), 4.44 (1H, apparent s, broad, 8-NHR), 3.11 (2H, d, *J* = 6.0 Hz, 3-NHC**H**_**2**_^t^Bu), 2.89 (3H, s, C**H**_**3**_SO_3_^−^), 2.65 (2H, d, *J* = 5.7 Hz, 8-NHC**H**_**2**_^t^Bu), 1.51 (3H, t, *J* = 7.1 Hz, NCH_2_C**H**_**3**_), 1.09 (9H, s, 3-NHCH_2_^**t**^**Bu**), 0.85 (9H, s, 8-NHCH_2_^**t**^**Bu**). HRMS (ESI^+^, m/z): found 470.3160. C_31_H_40_N_3_O ([M]^+^) requires 470.3166.

##### 3,8-Bis(neopentylamino)-2-hydroxy-5-(pentadeuteroethyl)-6-phenylphenanthridinium Mesylate **(d**_**5**_**-NeoOH)**

Fremy’s salt (22 mg, 0.09 mmol, 4.0 eq.) was dissolved in 10 mL of phosphate buffer (pH 7.4), through which argon was bubbled during 10 min before addition of salt. Freshly prepared ***d***_***5***_**-NeoH** (10 mg, 0.02 mmol, 1.0 eq.), which was used immediately after its synthesis, was dissolved in 0.5 mL of Et_2_O, transferred to the buffer and the mixture was stirred at RT under argon for 1 h in the dark. The solution was extracted with CHCl_3_ (3 × 10 mL), the organic layers combined, dried over MgSO_4_, filtered and concentrated under reduced pressure to give a raspberry red foam. The crude mixture was purified using preparative HPLC method by separate injections of 5 mg crude for each purification. Gradient elution of buffers A and B was from 58:42 to 50:50 over 45 min. Pure fractions were collected and combined. Brine was added, the solution was extracted with CHCl_3_, dried over anhydrous MgSO_4_, filtered and concentrated under reduced pressure to give ***d***_***5***_**-NeoOH** as a chloride salt. The solid was ion exchanged to the mesylate form [IRA 401 resin in mesylate form, loaded and eluted in MeOH-H_2_O (1:1)], furnishing **mesylate *d***_***5***_**-NeoOH** as a raspberry red glass (4.5 mg, 47%). t_R_ = 12 min. δ_H_ (500 MHz, CDCl_3_): 8.27 (1H, d, *J* = 9.2 Hz, H-10), 7.99 (1H, apparent s, broad, H-1), 7.71-7.64 (3H, m, Ph), 7.49-7.36 (2H, m, Ph), 7.34 (1H, d, *J* = 9.3 Hz, H-9), 6.62 (1H, s, H-4), 6.50 (1H, apparent s, broad, 3-N**H**R), 5.86 (1H, d, *J* = 2.2 Hz, H-7), 4.34 (1H, apparent s, broad, 8-NHR), 3.11 (2H, d, *J* = 5.9 Hz, 3-NHC**H**_**2**_^t^Bu), 2.86 (3H, s, C**H**_**3**_SO_3_^-^), 2.67 (2H, d, *J* = 5.8 Hz, 8-NHC**H**_**2**_^t^Bu), 0.95 (9H, s, 3-NHCH_2_^**t**^**Bu**), 0.89 (9H, s, 8-NHCH_2_^**t**^**Bu**). HRMS (ESI^+^, m/z): found 475.3457. C_31_H_35_D_5_N_3_O ([M]^+^) requires 475.3480.

##### d_15_-MitoNeo Dimer

Potassium hexacyanoferrate (39 mg, 0.12 mmol, 10.0 eq.) was added to a solution of *d*_*15*_-MitoNeoD (obtained from the reduction of MitoNeo (12 mg, 0.012 mmol) as described above) in MeCN (2 mL) and H_2_O (2 mL). The solution was stirred overnight then extracted into CH_2_Cl_2_ (2 × 10 mL). The combined organic layers were washed with brine (3 × 50 mL), dried over anhydrous Na_2_SO_4_ and concentrated under vacuum to give the dimer as a purple glass (8 mg, 67%, ∼85% purity by HPLC-UV, RT 19.2 min). HPLC analysis was conducted on a Shimadzu Prominence HPLC using a Phenomenex Kinetix 5μ EVO C-18 (250 mm x 4.6 mm) column eluting 30% MeCN in 0.1% TFA increasing to 80% MeCN over 30 min. HRMS (ESI^+^, m/z): found 392.7760. C_106_H_92_D_30_N_6_P_2_^4+^ (M^4+^) requires 392.7766. Isotope pattern consistent with structure.

##### Structural Assignment of MitoNeoOH and NeoOH

MitoNeoOH and NeoOH have been assigned as the 2-hydroxy derivatives (Figure 1A). This was done by first assigning the ^1^H NMR spectrum of Neo and then using this assignment to determine the position of substitution in NeoOH and by analogy MitoNeoOH. The assignment of Neo was carried out by NMR experiments on the triflate salt, while those on NeoOH used the chloride salt and those on MitoNeoOH on the mesylate salt. There are small differences in the chemical shifts and resolution of NMR signals for Neo salts depending on the counterion (triflate, mesylate or chloride) and concentration, but the overall pattern and critical upfield shift of H-7 (see below) is present in all. The triflate, mesylate and chloride salts could easily be interconverted by ion exchange and the salt with the best resolved signals in the NMR spectra were used in each case. The fully assigned ^1^H NMR spectrum of Neo is shown in Figure S4E. The basis of this assignment will be explained step-wise using the atom numbering shown in Figure S4F.

The chemical shifts, integrations and the matching coupling constants (7.1 Hz), allow the assignment of the 3H *t* at 1.54 ppm to the three hydrogen atoms attached to C12 (i.e. H12) and the 2H *q* at 4.78 ppm (expansion in Figure S4G) to the two hydrogen atoms attached to C11 (i.e. H11). This is also clear in the COSY spectra.

HSQC allowed the assignment of C11 to the signal at 49.5 ppm and C12 to the signal at 14.5 ppm by correlation to H11 and H12. The two NH protons were also assigned since they show no correlation in the HSQC spectrum. Differentiating between the two NH’s and the shown assignment to C8-NHR and C3-NHR (where R = neopentyl) was achieved below.

In the HMBC, the H11 *q* signal showed correlation to C12, plus correlation to quaternary carbons at 134.8 and 157.4 ppm. These are therefore C4a and C6. At this stage the assignment of C6 to the signal at 157.4 ppm rather than 134.8 ppm is made on chemical shift alone, and this assignment is confirmed below.

The H1-H2-H4 and H7-H9-H10 spins systems were identified by COSY. The final assignment of these was achieved using the 2H *dd J* = 7.6, 1.6 Hz at 7.41 ppm, which was reliably assigned to H2” based on integration, multiplicity, coupling constants and the COSY. With H2” assigned, the HMBC correlations H2”-C6(157.4 ppm)-H7 and H11-C4a(134.8 ppm)-H1 confirmed the identities of the spin systems. With H7 established, C7 was assigned to the signal at 105.1 ppm using HSQC, and HMBC correlation from C7 used to assign the C8-NH.

Above, we have outlined the steps used to assign the distinctive upfield signal at 6 ppm to H7 in Neo, and hence the H7-H9-H10 spin system. This upfield signal is also found in the ^1^H NMR spectra of other *N*-alkyl 6-phenylphenanthridinium salts in the literature and has consistently been assigned to H7. (Kreishman et al., 1971, Luedtke et al., 2005) It is also present in NeoOH at 5.97 ppm (chloride, 5.91 ppm mesylate). Thus, COSY confirms the H7-H9-H10 (5.97, 7.33 and 8.51 ppm) spin system is intact in NeoOH and the multiplicities and coupling constants of *J*_9,10_ = 9.0 Hz and *J*_7,9_ = 2.0 Hz are consistent with this. On the other hand, there are two singlets in the ^1^H NMR spectrum of NeoOH, which have no COSY correlations, so can be assigned to the *para*-related protons H1 (8.66 ppm) and H4 (6.70 ppm). There is little change to the protons of the 6-phenyl ring (3H, 7.69-7.76 ppm and 2H, 7.41-7.47 ppm) as would be expected from a distant change in structure, and there is no signal that could be assigned to a hydrogen atom attached to C2.

The signals for the phenanthridinium ring system in the spectra of MitoNeoOH and NeoOH with the same counterion are very similar. Detailed analysis of MitoNeoOH was carried out using the NMR spectra of the mesylate because this was the well resolved. The distinctive upfield shift of H7 allows the COSY (Figure S4H, atom numbering in Figure S4I) to confirm that the H7-H9-H10 spin system is intact and the assignment of two singlets shown in red to H1 and H4, demonstrating that the addition regiochemistry is the same for both NeoOH and MitoNeoOH.

### Quantification and Statistical Analysis

Statistical analysis was performed using GraphPad Prism. Statistical values including the exact n, the test used and the statistical significance are also reported in the Figure Legends.

## Author Contributions

R.C.H., A.L., M.M.S., A.G.C., and M.P.M. devised the method. M.M.S., A.G.C., and S.T.C. carried out the chemical syntheses. A.L. and T.A.P. developed the mass spectrometry analysis. S.A. carried out mass spectrometry analyses. A.L., T.A.P., J.N.C., H.A.P., and T.P.B. carried out many of the mitochondrial and cell analyses. P.Y. and R.F.A. carried out the pulse radiolysis experiments. S.V. and A.R.H. carried out image analysis. V.R.P., J.F.M., and T.K. carried out the mouse experiments. H.M.S. carried out the computer modeling. M.P.M., A.L., A.M.J., R.F.A., and R.C.H. wrote the paper. All authors reviewed the manuscript.

## Supporting Citations

The following references appear in the Supplemental Information: Doskey et al., 2015, Reily et al., 2013.

## Supplemental Information

Document S1. Figures S1–S7 and Table S1

Document S2. Article plus Supplemental Information
