# Supplementary material for: MitoNeoD: A Mitochondria-Targeted Superoxide Probe
Source: Cell Chem Biol. 2017 Oct 19;24(10):1285–1298.e12. doi: 10.1016/j.chembiol.2017.08.003 (PMC6278870; doi:10.1016/j.chembiol.2017.08.003)
Supplement: Document S1. Figures S1–S7 and Table S1 [file mmc1.pdf]

Cell Chemical Biology, Volume *24*

Supplemental Information

MitoNeoD: A Mitochondria-Targeted Superoxide Probe

Maria M. Shchepinova, Andrew G. Cairns, Tracy A. Prime, Angela Logan, Andrew M. James, Andrew R. Hall, Sara Vidoni, Sabine Arndt, Stuart T. Caldwell, Hiran A. Prag, Victoria R. Pell, Thomas Krieg, John F. Mulvey, Pooja Yadav, James N. Cobley, Thomas P. Bright, Hans M. Senn, Robert F. Anderson, Michael P. Murphy, and Richard C. Hartley

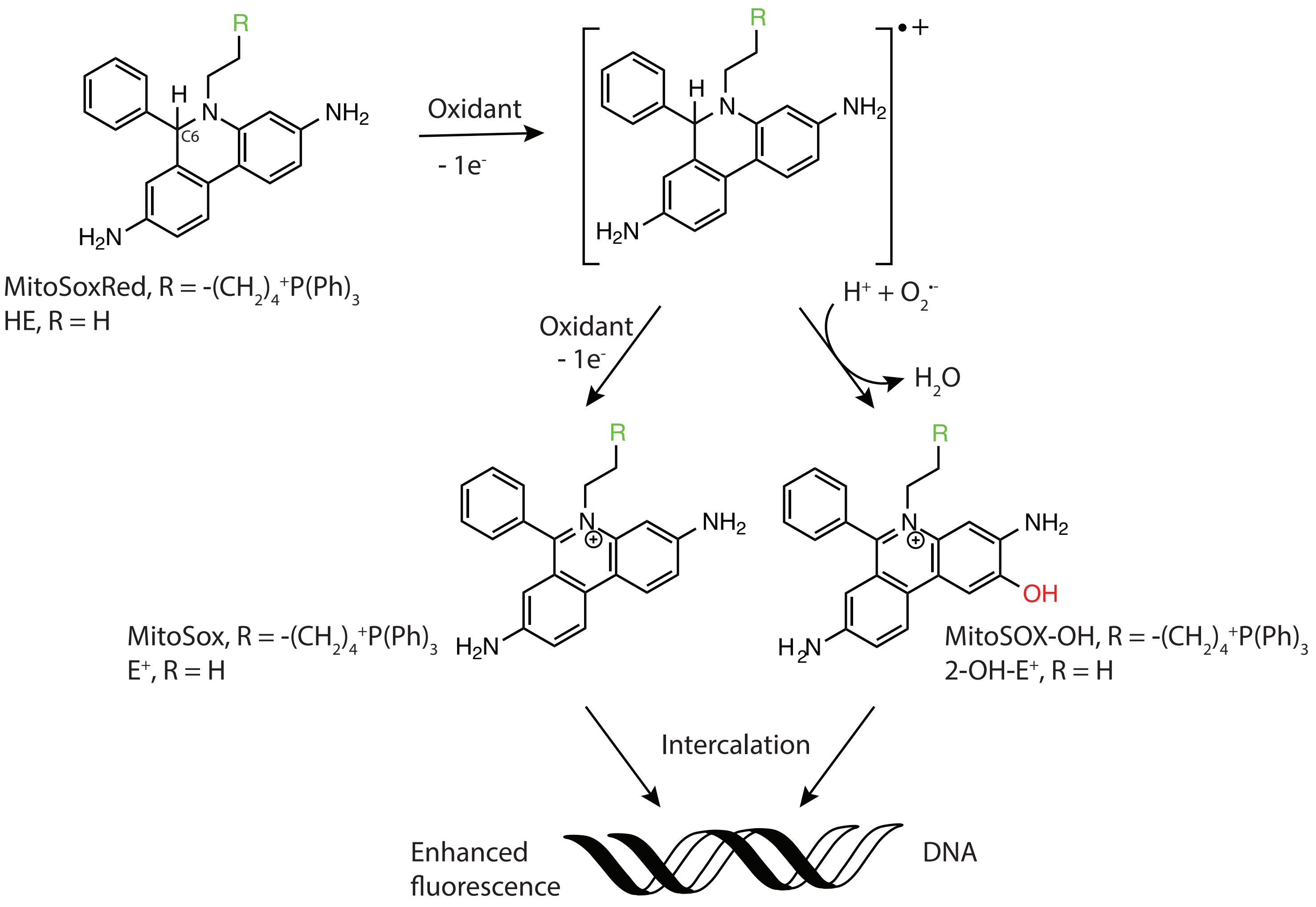

Figure S1. Reactions of HE and MitoSOX Red (*Related to Figure 1*)
Hydroethidine (HE) and MitoSOX Red (=MitoHE) react with $O_2^{\bullet -}$ to form a radical cation that then reacts with $O_2^{\bullet -}$ to form a hydroperoxide adduct that rearranges to a quinone imine form and then to a 2-hydroxy derivative. However, other one-electron oxidants can also generate the radical cation that can be further oxidized, or disproportionate to E^+, as well as undergoing other side reactions to form dimeric products (not shown). The intrinsic fluorescence of these products is greatly enhanced by their intercalation into DNA.

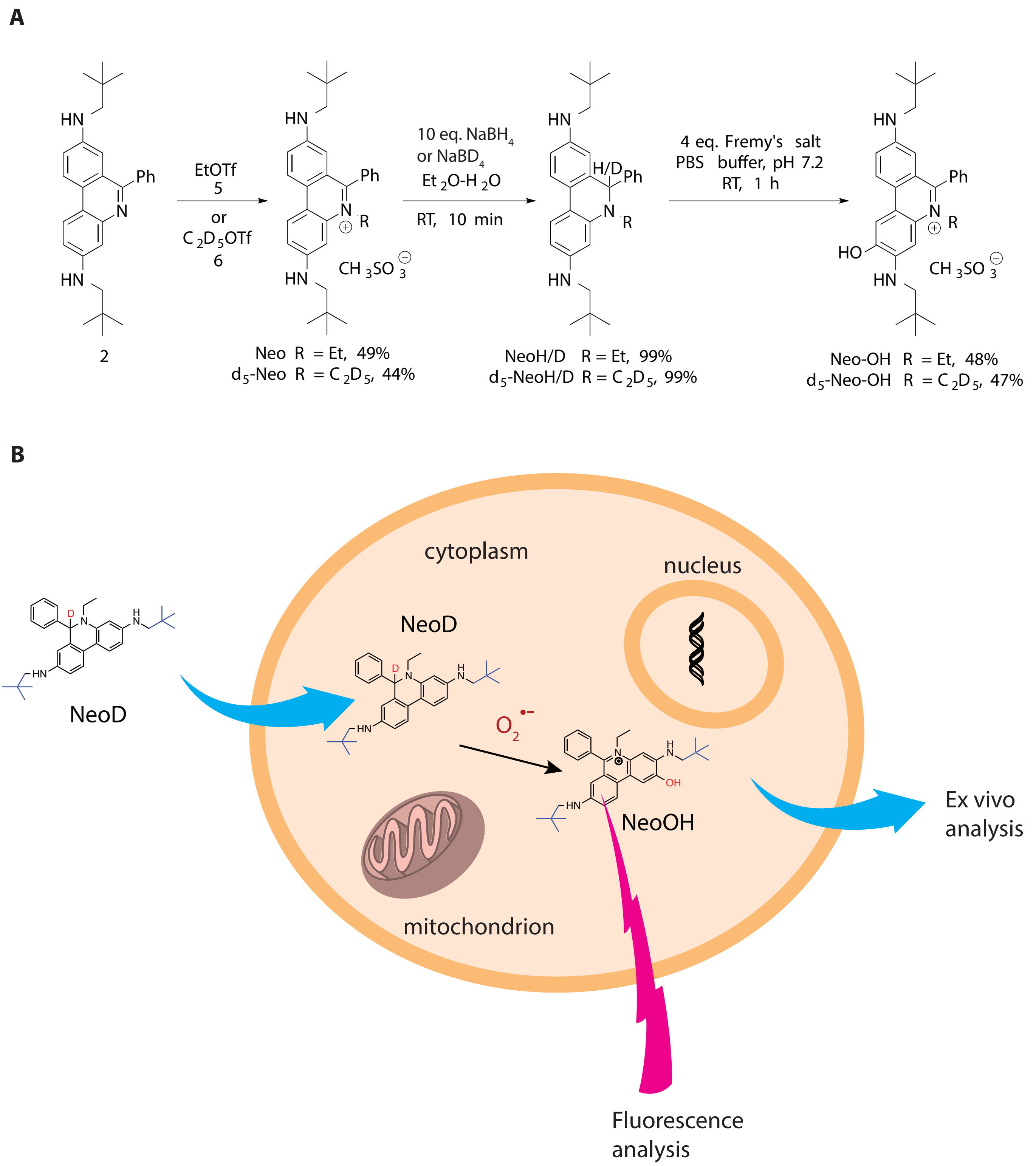

Figure S2. Synthesis and use of Neo to Assess Cellular $O_2^{\bullet-}$ (*Related to Figure 1*)

(A) Synthesis of Neo, NeoH/D and NeoOH. Ethylation of the bis(neopentyl) derivative **2** was achieved with ethyl triflate **5** to give Neo as the mesylate salt. d_5-Neo was prepared from **2** with d_5-ethyl triflate **6**. Reduction of Neo in a two-phase water-diethyl ether mixture under argon by $NaBH_4$/$NaBD_4$ gave NeoH/D. NeoOH was prepared by reaction of NeoH/D with Fremy's salt (potassium nitrosodisulfonate). Et = ethyl, Me = methyl, Ph = phenyl.

(B) Reactions of Neo in cells and *in vivo*. NeoD within cells is not taken up by mitochondria and therefore reacts with superoxide in the cell. The $O_2^{\bullet-}$-dependent reaction forms NeoOH, while, the non-specific oxidation product Neo is also formed. NeoOH formation in cells can be detected within cells by confocal microscopy and following extraction by HPLC or LC-MS/MS.

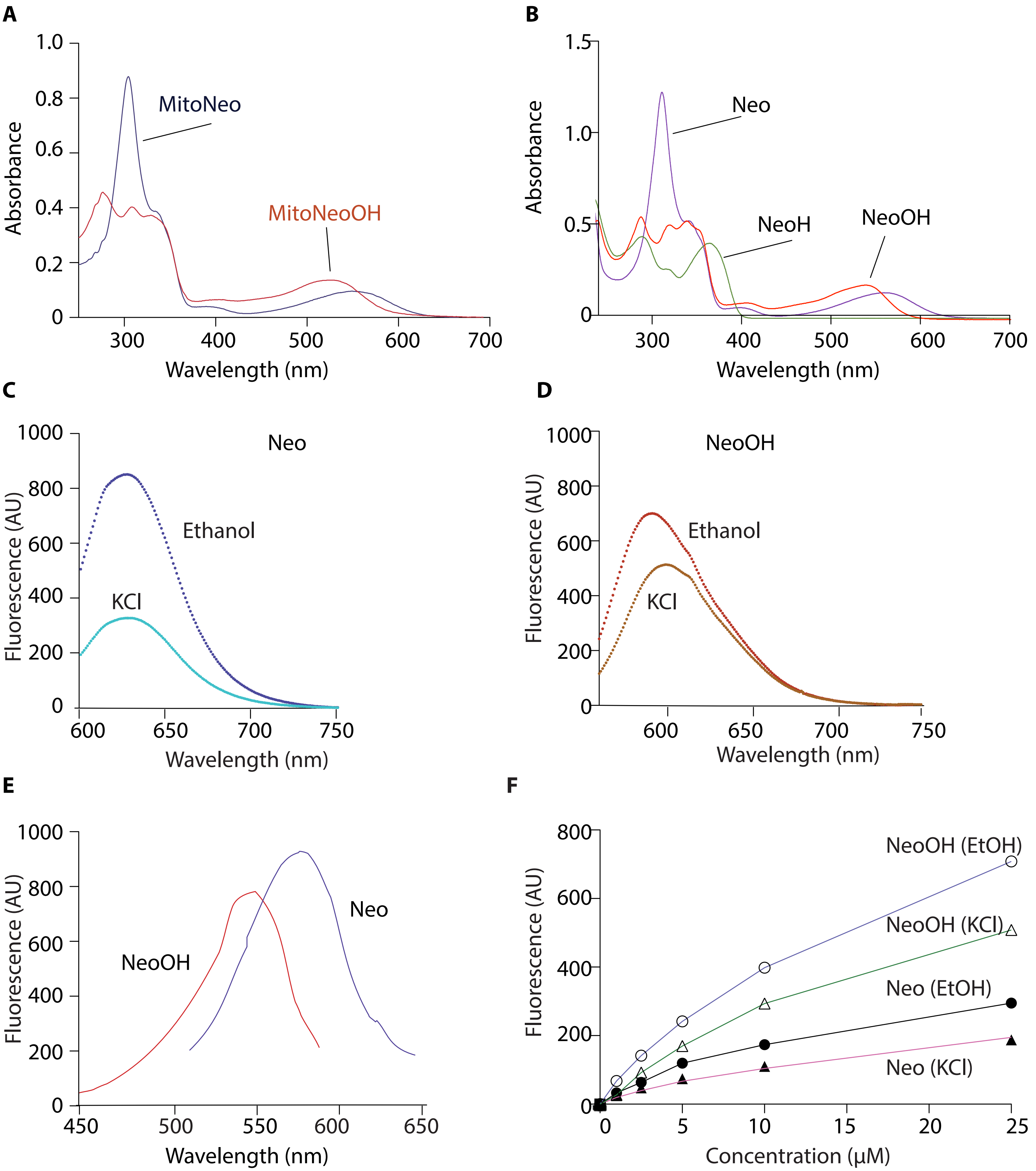
A
MitoNeo
MitoNeoOH
Absorbance
Wavelength (nm)
B
Neo
NeoH
NeoOH
Absorbance
Wavelength (nm)
C
Neo
Ethanol
KCl
Fluorescence (AU)
Wavelength (nm)
D
NeoOH
Ethanol
KCl
Fluorescence (AU)
Wavelength (nm)
E
NeoOH
Neo
Fluorescence (AU)
Wavelength (nm)
F
NeoOH (EtOH)
NeoOH (KCl)
Neo (EtOH)
Neo (KCl)
Fluorescence (AU)
Concentration (μM)

Figure S3. UV/visible Absorption and Fluorescent Properties of MitoNeo, Neo and Their Derivatives (*Related to Figure 2*)

(A) UV/vis absorption spectra of MitoNeo or MitoNeoOH in KCl buffer supplemented with 20% ethanol (all 25 μM).

(B) UV/vis absorption spectra of Neo, NeoH or NeoOH in ethanol (all 25 μM).

(C-E) Fluorescence spectra of Neo/NeoOH (25 μM of each) in ethanol or KCl buffer supplemented with 20% ethanol at RT. At these concentrations in KCl buffer NeoH caused cloudiness and light scattering.

(C) Emission fluorescence spectra of Neo in ethanol and in KCl buffer. Excitation wavelengths: 577 nm (ethanol), 573 nm (KCl).

(D) Emission fluorescence spectra of NeoOH in ethanol and in KCl buffer. Excitation wavelengths: 550 nm (ethanol), 548 nm (KCl).

(E) Excitation fluorescence spectra of Neo and NeoOH in ethanol. Emission wavelengths: 628 nm (ethanol), 591 nm (KCl).

(F) Concentration dependence of fluorescence of NeoOH and Neo at the excitation and emission maxima of NeoOH in ethanol (550/591 nm) or KCl buffer (548/599 nm).

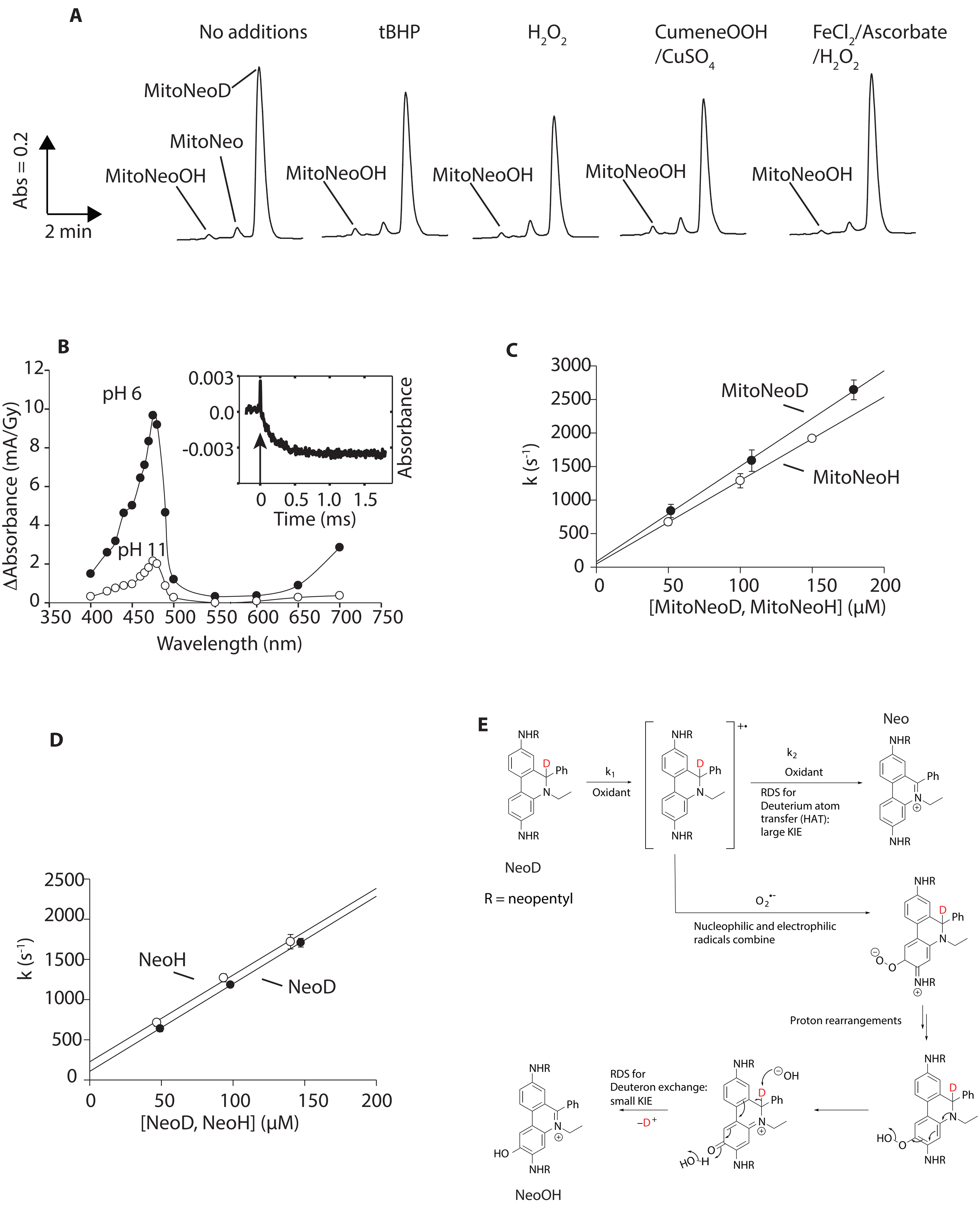
A
No additions
tBHP
H2O2
CumeneOOH /CuSO4
FeCl2/Ascorbate /H2O2
MitoNeoD
MitoNeo
MitoNeoOH
Abs = 0.2
2 min
B
pH 6
pH 11
ΔAbsorbance (mA/Gy)
Wavelength (nm)
Time (ms)
Absorbance
C
MitoNeoD
MitoNeoH
k (s-1)
[MitoNeoD, MitoNeoH] (μM)
D
NeoH
NeoD
k (s-1)
[NeoD, NeoH] (μM)
E
NeoD
R = neopentyl
k1
Oxidant
k2
Oxidant
RDS for Deuterium atom transfer (HAT): large KIE
Neo
O2•−
Nucleophilic and electrophilic radicals combine
Proton rearrangements
RDS for Deuteron exchange: small KIE
−D+
NeoOH
NHR
Ph
D

Figure S4. ***In vitro*** **Oxidation of NeoH/D by $O_2^{\bullet -}$ Measured by Fluorescence and Structural Assignment of NeoOH and MitoNeoOH by ^{1}H NMR (cont.** ***Related to Figure 3*****)**

(A) Emission fluorescence spectra over time. NeoH (10 μM) was incubated at 37°C in KCl buffer (pH 7.2) supplemented with 20% EtOH with 1 mM HX and 5 mU/mL XO. Emission spectra were acquired at the indicated times using an excitation maximum of NeoOH (548 nm). The emission maximum for NeoOH in KCl (599 nm) is indicated.

(B) Excitation fluorescence spectra over time. NeoH (10 μM) was incubated at 37°C in KCl buffer (pH 7.2) supplemented with 20% EtOH with 1 mM HX and 5 mU/ml XO. The excitation spectrum was assessed at various times using an emission wavelength of 599 nm. The excitation maximum for NeoOH in KCl (548 nm) is indicated.

(C, D) Time course of reaction of NeoH or NeoD with $O_2^{\bullet -}$. NeoH or NeoD (10 μM) was incubated at 37°C in KCl buffer (pH 7.2) supplemented with 20 % EtOH with 1 mM HX and 5 mU/ml XO. Excitation and emission wavelengths were 548 nm and 599 nm respectively, the maxima for NeoOH in KCl buffer. Where indicated, SOD (10 μg/mL) or catalase (50 U/mL) were added.

(E – I) Structural assignment of NeoOH and MitoNeoOH.

(E) The fully assigned ^{1}H NMR spectrum of Neo triflate is shown.

(F) Structure of Neo showing numbering of carbon atoms used in the discussion.

(G) Expansion of the signal at 4.78 ppm from (E).

(H) Aromatic region in COSY spectrum of MitoNeoOH mesylate.

(I) Structure of MitoNeoOH showing numbering.

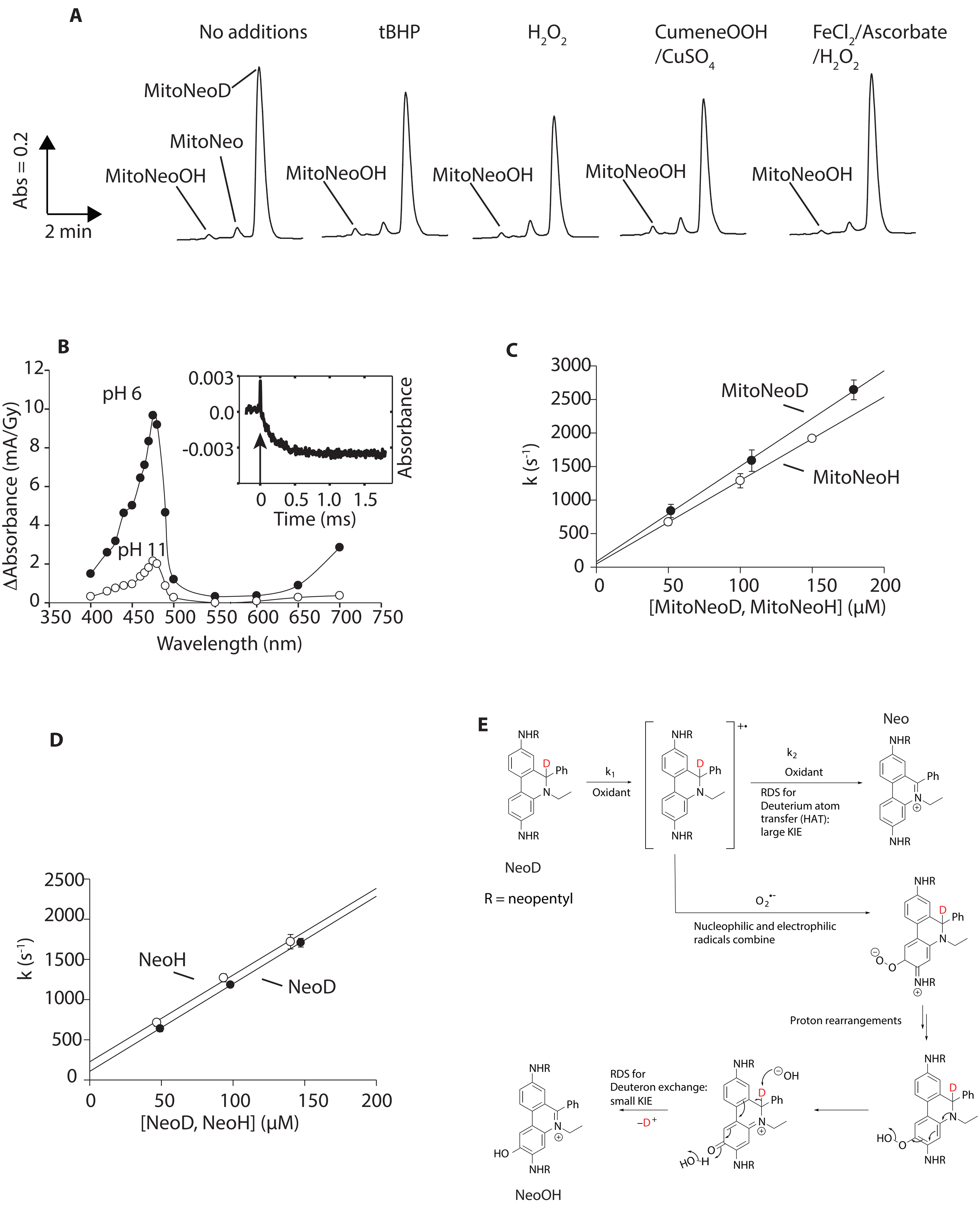
A
No additions
tBHP
H_2O_2
CumeneOOH
$/CuSO_4$
$FeCl_2$/Ascorbate
$/H_2O_2$
MitoNeoD
MitoNeo
MitoNeoOH
Abs = 0.2
2 min
B
pH 6
pH 11
ΔAbsorbance (mA/Gy)
Wavelength (nm)
Absorbance
Time (ms)
C
MitoNeoD
MitoNeoH
k (s^{-1})
[MitoNeoD, MitoNeoH] (μM)
D
NeoH
NeoD
k (s^{-1})
[NeoD, NeoH] (μM)
E
NeoD
R = neopentyl
k_1
Oxidant
k_2
Oxidant
RDS for
Deuterium atom
transfer (HAT):
large KIE
Neo
$O_2^{\bullet -}$
Nucleophilic and electrophilic
radicals combine
Proton rearrangements
RDS for
Deuteron exchange:
small KIE
$-D^+$
NeoOH

Figure S5. Reactions of MitoNeoH/D with Various ROS Analysed by RP-HPLC and with $O_2^{\bullet-}$ Analysed by Pulse Radiolysis (cont. *Related to Figure 4*)

(A) Reaction of MitoNeoD with different ROS analysed by RP-HPLC. MitoNeoD (20 nmol) was incubated for 40 min in 250 μl KCl buffer (pH 7.2) at 37 ºC with either no additions, 500 μM *tert*-butylhydroperoxide (tBHP), 100 μM H_2O_2, 100 μM cumene hydroperoxide (CumeneOOH) and 25 μM $CuSO_4$ or 40 μM $FeCl_2$, 1 mM ascorbate and 100 μM H_2O_2. After 30 min samples were snap frozen and later analyzed by RP-HPLC as described, assessing MitoNeo and MitoNeoOH formation at 220 nm. Incubation with 100 μM peroxynitrite or with 500 μM DETA-NONOate (to generate NO), under the same conditions as above did not generate MitoNeoOH or MitoNeo, but other peaks were evident, presumably due to nitration under these conditions (data not shown).

(B) Spectral changes measured 2 ms after pulse radiolysis (3 Gy in 200 ns) of air:N_2O (1:1) saturated solutions of water:ethanol (1:1) containing NeoD (150 μM) at pH 6, ●, and at pH 11, ○. Spectra are corrected for subsequent decay. Insert: change in transmittance at 475 nm at pH 6 following pulse radiolysis (arrow).

(C) Dependence of the first-order rate constants for the formation of the absorption at 475 nm at pH 11 on the concentration of MitoNeoH, ○, and MitoNeoD, ●. Experimental conditions as in (B).

(D) Dependence of the first-order rate constants for the formation of the absorption at 475 nm at pH 11 on the concentration of NeoH, ○, and NeoD, ●. Experimental conditions as in (B).

(E) Schematic for reaction of MitoNeoH/D with $O_2^{\bullet-}$ explaining the lack of an initial KIE. KIE, Kinetic isotope effect. RDS, rate determining step.

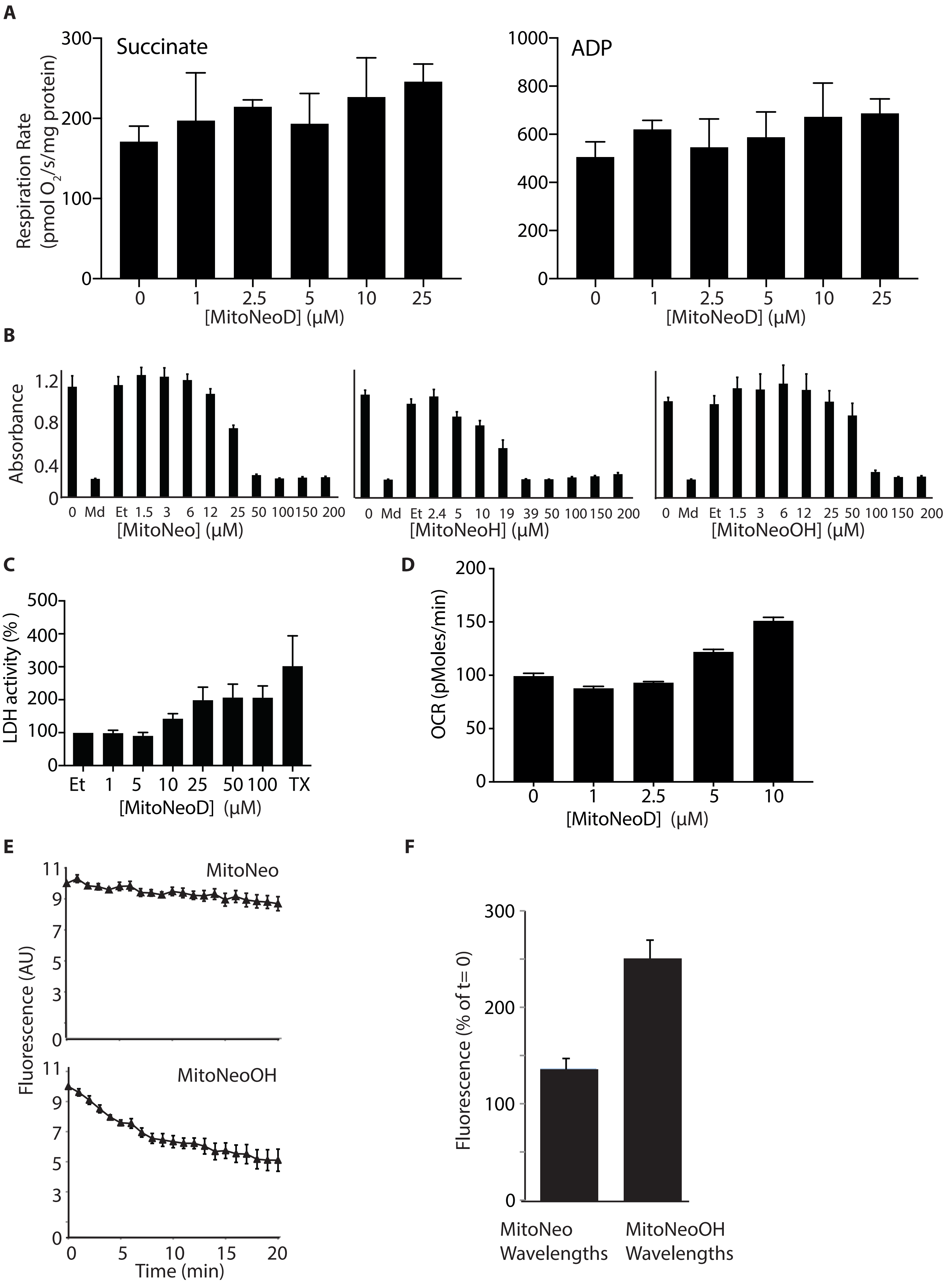

A
Succinate
ADP
Respiration Rate (pmol O2/s/mg protein)
0
100
200
300
200
400
600
800
1000
0
1
2.5
5
10
25
[MitoNeoD] (μM)
B
Absorbance
0
0.4
0.8
1.2
0 Md Et 1.5 3 6 12 25 50 100 150 200
[MitoNeo] (μM)
0 Md Et 2.4 5 10 19 39 50 100 150 200
[MitoNeoH] (μM)
0 Md Et 1.5 3 6 12 25 50 100 150 200
[MitoNeoOH] (μM)
C
LDH activity (%)
0
100
200
300
400
500
Et 1 5 10 25 50 100 TX
[MitoNeoD] (μM)
D
OCR (pMoles/min)
0
50
100
150
200
0
1
2.5
5
10
[MitoNeoD] (μM)
E
MitoNeo
MitoNeoOH
Fluorescence (AU)
0
3
5
7
9
11
0
5
10
15
20
Time (min)
F
Fluorescence (% of t= 0)
0
100
200
300
MitoNeo Wavelengths
MitoNeoOH Wavelengths

Figure S6. Interactions of MitoNeo and its Derivatives With Mitochondria and Cells (*Related to Figure 6*)
(A) Effect of MitoNeoD on respiration by isolated mitochondria. Rat liver mitochondria (1 mg protein/ml) were incubated in an Oroboros Oxygraph 2K respirometer in 2 mL buffer containing 120 mM KCl, 10 mM HEPES, 1 mM EGTA, 5 mM KH_2PO_4, pH 7.2, supplemented with rotenone (4 μg/ml) at 37˚C were incubated with indicated concentrations of MitoNeoD or ethanol carrier. Then succinate (10 mM) was added and coupled respiration was measured. After this ADP (75 μM) was added and phosphorylating respiration was measured. Data are means ± SD of 3 - 4 independent mitochondrial preparations.
(B) Toxicity of MitoNeo, MitoNeoH and MitoNeoOH on C2C12 cells. C2C12 cells were seeded in a 96 well plate at 10,000 cells/well, grown overnight and then various concentrations of the indicated compounds were added and compared with no additions or ethanol carrier (Et). Menadione (50 μM; Md) was used as a positive control for cell death. After incubation for 17.5 h cell viability was assessed by the MTS assay and the absorbance measured at 490 nm. Data are means ± SD for 8 wells.
(C) Toxicity of MitoNeoD on HeLa cells. HeLa cells (from the American Type Culture Collection) were seeded in a 96 well plate at 7,000 cells/well, grown overnight incubated at 37 ºC in a humidified atmosphere of 95 % air and 5 % CO_2 in Dulbecco's modified Eagle's medium (DMEM; Invitrogen) supplemented with 10 % (v/v) FBS and 100 U/ml penicillin and 100 μg/ml streptomycin. After washing the cells with phenol-red free DMEM, cells were incubated with various concentrations of MitoNeoD in phenol-red free DMEM for 4 h at 37 ºC. After the incubation release of lactate dehydrogenase (LDH) was measured using the Cytotoxicity Detection Kit (LDH) from Roche according to the manufacturer's instructions. Cells treated with 2% ethanol were set as 100% viability. Data are means ± SEM.
(D) Effect of MitoNeoD on cell respiration. C2C12 cells were plated at 10, 000 cells per well in complete growth medium and left to adhere at RT for 1 h, before incubation at 37˚C overnight in 95% air/5% CO_2. Then growth medium was replaced with HEPES buffered DMEM containing 0.2% BSA and cells were incubated at 37 °C for 40 min in air. MitoNeoD or ethanol carrier was added, incubated for 20 min at 37˚C and then oxygen consumption was measured using a Seahorse X96 extracellular flux analyser as described in (Reily et al. 2013). Data are means ± SEM for three independent experiments, in which each measurement was the mean of 10 replicates. Under these conditions 5 μM MitoNeoD increases basal respiration, presumably due to accumulation and uncoupling. This dose response is similar to a range of similar mitochondrial targeted TPP compounds (Reily et al. 2013). The effects impact at a lower concentration in this system compared to the toxicity assays shown in panels A – C. This is because of the disproportionately large accumulation of compound per cell due to the small number of cells and the large incubation volume (Doskey et al. 2015).
(E) Effect of FCCP addition after MitoNeo and MitoNeoOH accumulation. Cells were incubated with MitoNeo or MitoNeoOH for 10 min and the fluorescence in mitochondrial regions quantified as described in the legend to Figure 6B. Then FCCP (0.5 μM) was added and the change in fluorescence plotted against time. Data = means ± SD, n = 4.
(F) Change in fluorescence of MitoNeoD incubated with cells over time assessed at wavelengths optimised for MitoNeo, or for MitoNeoOH. C2C12 cells were incubated with MitoNeoD and the fluorescence was assessed at wavelengths optimised for the detection of MitoNeo or MitoNeoOH and again after 20 min incubation with menadione (50 μM). Fluorescence data obtained at 20 min are expressed as a percentage of the fluorescence at t = 0. Data = means ± SD, n = 4.

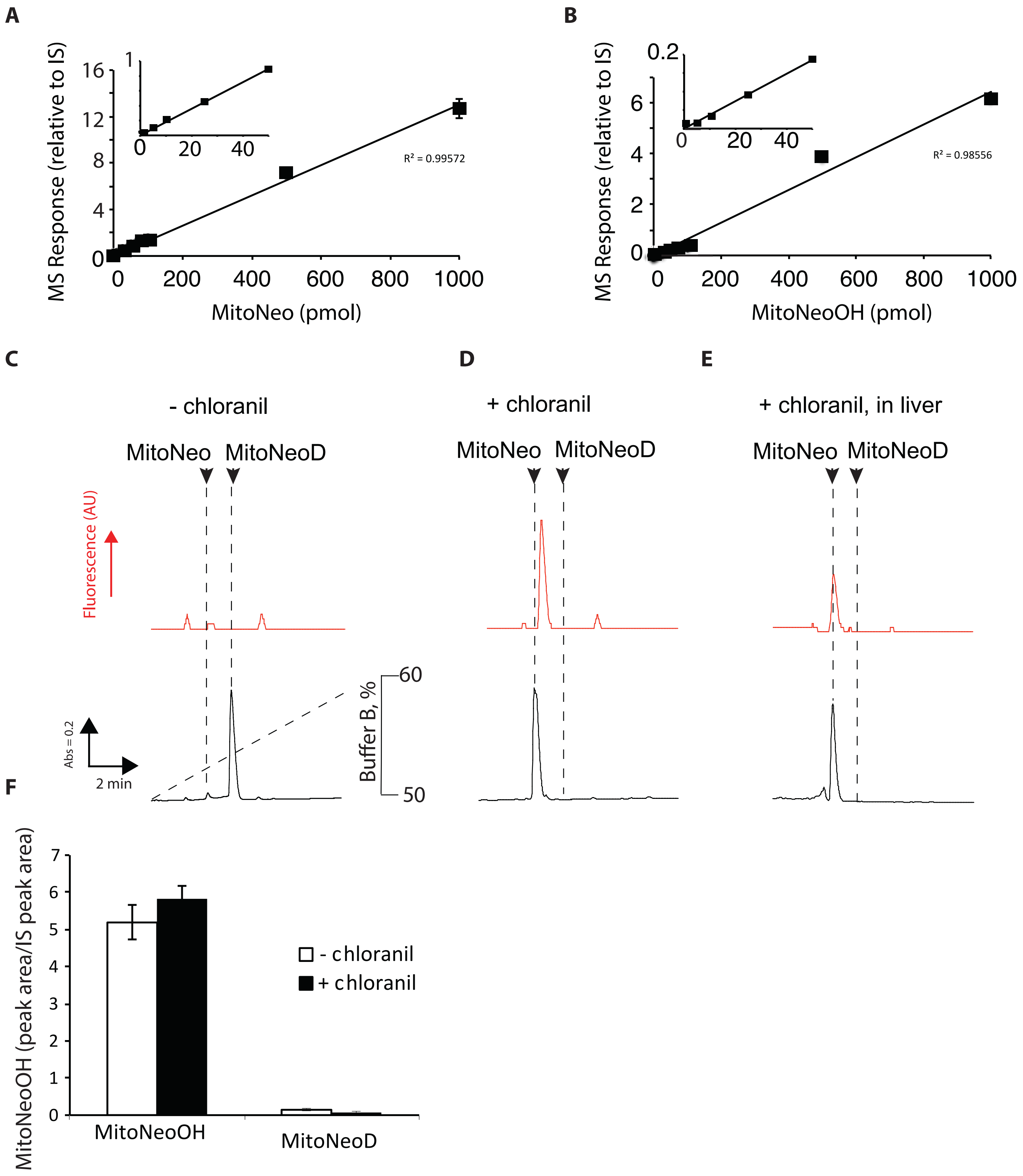
A
MS Response (relative to IS)
16
12
8
4
0
1
0
20
40
R² = 0.99572
0
200
400
600
800
1000
MitoNeo (pmol)
B
0.2
6
4
2
0
20
40
R² = 0.98556
MitoNeoOH (pmol)
C
- chloranil
MitoNeo
MitoNeoD
Fluorescence (AU)
Abs = 0.2
2 min
Buffer B, %
60
50
D
+ chloranil
MitoNeo
MitoNeoD
E
+ chloranil, in liver
MitoNeo
MitoNeoD
F
MitoNeoOH (peak area/IS peak area)
7
6
5
4
3
2
1
0
- chloranil
+ chloranil
MitoNeoOH
MitoNeoD

Figure S7. Mass Spectrometric Analysis and Interactions of MitoNeo and its Derivatives With Chloranil (*Related to Figure 7*)

(A, B) Typical standard curves for MitoNeo and MitoNeoOH. Standard curves are prepared in parallel with samples, with appropriate biological material spiked with a range of MitoNeo or MitoNeoOH concentrations and the deuterated ISs. Standard curves shown are prepared from liver. For this, sections of liver (50 mg wet weight) were homogenised and spiked with known amounts of either MitoNeo or MitoNeoOH, along with IS (100 pmol d_{15}-MitoNeo and 50 pmol d_{15}-MitoNeoOH). Standards were incubated with chloranil for 1 h, extracted with butanol/methanol, and MitoNeo and MitoNeoOH assessed by LC-MS/MS.

(C, D) Reaction of chloranil with MitoNeoD. MitoNeoD (40 μM) in 250 μL KCl buffer was incubated for 30 min alone (C) or with chloranil (80 μM) at 37°C with shaking at 1,000 rpm. Then the samples were diluted to 20 %ACN/0.1 % TFA and analysed by RP-HPLC as described in the legend to Figure 4.

(E) Reaction of chloranil with MitoNeoD in a liver homogenate. MitoNeoD (40 μM) in 250 μL KCl buffer was homogenised with rat liver (50 mg wet weight) as described for LC-MS/MS and incubated with chloranil (80 μM) as in (C, D) above. Then the sample was extracted into 1 mL butan-2-ol/methanol (3:1) sonicated for 1h at RT, dried in a speed vac then re-suspended in 400 μL 40% methanol/0.1% FA mixed with 1 mL 25% ACN/0.1% TFA and analysed by RP-HPLC as described in the legend to Figure 4.

(F) Reaction of chloranil with MitoNeoOH and MitoNeoD in a liver homogenate analysed by LC-MS/MS. MitoNeoOH (40 μM) or MitoNeoD (40 μM) were incubated in a liver homogenate as described in (E) above. Then the sample was spiked with d_{15}-MitoNeoOH as an internal standard and the concentration of MitoNeoOH was determined by LC-MS/MS. Data are presented as the area under the peak relative to IS and are the means ± SD, n = 3.

Table S1. Absorption Maxima and Extinction Coefficients; Fluorescence Excitation and Emission Maxima (Related to Figure 2)

	Absorption		Fluorescence			
	Ethanol	KCl	Ethanol		KCl	
	λ_{max} [nm] (ε [mM^{-1} cm^{-1}])		Excitation λ_{max} [nm]	Emission λ_{max} [nm]	Excitation λ_{max} [nm]	Emission λ_{max} [nm]
Compound						
Neo	557 (5.8)	549 (5.7)	577	628	573	630
NeoOH	535 (7.8)	525 (4.2)	550	591	548	599
NeoH/D	358 (16.9)	379 (13)				
MitoNeo	561 (6.4)	550 (3.8)	561	628	566	636
MitoNeoOH	532 (7.3)	528 (5.5)	549	591	544	605
MitoNeoH/D	358 (20)	359 (8.7)	368	422	363	412
